# The illusion of internal models in biological movement

**DOI:** 10.1007/s00421-025-05963-3

**Published:** 2025-08-27

**Authors:** Madhur Mangalam

**Affiliations:** Department of Biomechanics, University of Nebraska at Omaha, Omaha, NE 68182 USA

**Keywords:** Sensorimotor control, Internal models, Representation, Ecological psychology, Dynamical systems, Embodied cognition

## Abstract

The concept of internal models dominates contemporary theories of sensorimotor control, with researchers across neurosciences, specifically motor control, routinely explaining observed behaviors through computational representations that supposedly exist within the nervous system. In this perspective, I present a critical examination of internal model frameworks in sensorimotor control. I argue that representational approaches mischaracterize biological systems for several fundamental reasons: (1) Internal models require homuncular interpreters, creating infinite regress problems; (2) The purported neural implementations of internal models remain empirically elusive despite decades of research; (3) Biological movement systems exhibit multiscale, nonlinear, and non-Gaussian dynamics that fundamentally defy reduction to conventional computational representations; (4) Internal model frameworks implicitly depend on Cartesian dualism through their separation of the “controller” and “controlled;” (5) The framework is methodologically circular and largely unfalsifiable as virtually any behavior can be retroactively modeled as implementing some internal representation; and (6) Alternative frameworks based on ecological dynamics and self-organization can account for adaptive behavior without invoking representational assumptions. Instead of representational models, I propose that sensorimotor control emerges from the dynamic coupling between the organism and the environment across multiple spatial and temporal scales. By moving beyond the internal model paradigm, sensorimotor neuroscience can develop more powerful explanatory frameworks that better capture the emergent, context-sensitive properties of biological movement without invoking physiologically intractable computational metaphors.

## The ubiquity of internal model thinking

The seduction of internal models lies not in what they explain, but in how they flatter our engineering instincts—projecting computational order onto biological chaos and mistaking our metaphors for mechanisms.

Sensorimotor control theory is deeply permeated with the concept of internal models. When a behavior is explained as utilizing neural representations of bodily dynamics, environmental properties, or the causal structure of the world, the so-called “internal model” is implicitly or explicitly invoked. This approach has become so ubiquitous that it appears across neuroscience, psychology, robotics, and biomechanics (Ito 2008; Kawato 1999; Schaal and Schweighofer 2005; Shadmehr and Krakauer 2008; Wolpert et al. 1998; Wolpert and Ghahramani 2000. Internal model frameworks involve both a computational and an empirical component. The computational component addresses the algorithmic implementation: how might neural circuits compute, store, and utilize representations of the body and world to enable effective action? Though computationally intricate, formal implementations of internal models are well defined within mathematical frameworks of control theory, Bayesian inference, neural networks, and the like. However, the real challenge lies in the empirical validity: after 5 decades and billions in research funding, do these models accurately describe how biological systems actually control movement? The answer, as I demonstrate here, is a resounding no.

While this critique is centered on human sensorimotor control, many of the arguments—particularly those concerning representational ambiguity, dynamical coordination, and ecological interaction—extend to nonhuman animals as well—which have also been purported to have some sort of internal models (Dauzere-Peres and Wystrach 2024; Kim et al. 2015; Lisberger 2009; Ramnani 2006; Webb 2004). There exists a substantial body of research on perceiving–acting systems in animals (e.g., insects, birds, fish, and nonhuman primates) that supports non-representational accounts of behavior grounded in ecological dynamics and direct sensorimotor coupling (Berger Dauxère et al. 2021; Fragaszy et al. 2021; Wagman et al. 2019). Although a full treatment of this literature is beyond the present scope, it offers important convergent evidence for the claims developed here and underscores the broader applicability of non-representational frameworks across species.

The ascendancy of internal model theories traces to the convergence of cybernetics, control engineering, and early cognitive neuroscience in the mid-to-late 20th century. Concepts developed by Norbert Wiener (cybernetics; Wiener 1948), Rudolf Kalman (state estimation and Kalman filtering; Kalman 1960), and David Marr (computational levels of analysis; Marr 1982) laid the groundwork for viewing the brain as a control-theoretic system—an inference engine that simulates and predicts its environment. As computational resources grew and robotics matured, the analogy between biological and engineered systems solidified. Neuroscientists increasingly import experimental and analytical methods designed for programmable artifacts into explanations of sensorimotor behavior, without reconsidering their ontological fit. What emerged was not an inevitable scientific trajectory, but a contingent, culturally shaped framework—one whose influence owed as much to the technological esthetics of the 20th century as to biological observation.

**Fig. 1:**
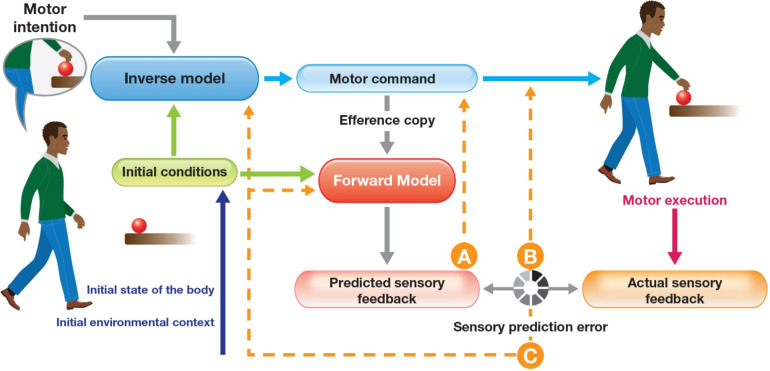
Internal model frameworks in sensorimotor control. This architecture proposes that voluntary movement is orchestrated through a computational cycle involving inverse and forward models. The inverse model converts intended goals and initial states into specific motor commands, while the forward model generates anticipated sensory consequences from a copy of those commands. These anticipations are used to shape motor output in advance of actual movement (A). Once the action begins, the nervous system compares expected and incoming sensory information; discrepancies between them—so-called prediction errors—may trigger mid-movement corrections, assuming the action unfolds over a sufficient duration (B). Such discrepancies are also believed to recalibrate the internal mappings that govern future actions, tuning both forward and inverse models for improved alignment (C). As examined throughout this manuscript, these frameworks rest on hypothetical internal representations, presume a top-down control structure, and rely on an idealized error-detection mechanism. They also inherit longstanding conceptual difficulties, including the homunculus fallacy, the problem of symbol grounding, and circular explanatory logic. These challenges undermine the plausibility of internal model theory as a biological account of motor control, prompting the need for alternatives rooted in bodily interaction, dynamical processes, and ecological context

At its core, internal model frameworks posit that the brain constructs and maintains internal representations of the body and environment, which are then used to predict the sensory consequences of motor commands and to plan corrective actions. These models are often conceptualized in terms of forward and inverse functions—one predicting sensory outcomes from motor commands, the other computing motor commands needed to achieve desired outcomes (**Fig.** 1). While these formulations are computationally elegant and align well with engineering control theory, they rest on the assumption that the nervous system operates as a predictive, representational system analogous to a digital computer. These assumptions grant internal models a powerful explanatory gloss but also expose them to serious philosophical and empirical scrutiny. Crucially, internal models are not directly observable; their existence is inferred from behavior, making the framework vulnerable to circular reasoning and post hoc rationalization.

Three fundamental issues undermine the empirical application of internal model theories:

1. Any behavior can be modeled as utilizing internal representations if given enough degrees of freedom (Barrett 2011; Chemero 2009).
2. The theoretical concept of representation remains poorly defined, with proponents shifting between literal and metaphorical interpretations as empirical challenges arise (Bechtel 1998; Haselager et al. 2003; Ramsey 2007).
3. The neural implementations of purported internal models remain elusive despite decades of dedicated research (Ebner et al. 2011; Krakauer et al. 2017; Loeb 2012).

Despite these foundational problems, internal model frameworks continue to dominate as the prevailing explanatory paradigm in sensorimotor control research (Franklin and Wolpert 2011; Scott 2004). This persistence is driven less by empirical success than by historical momentum and deeply ingrained commitments to computational interpretations of biological function. The allure of internal models is powerful—they promise to tame the overwhelming complexity of movement through concise and elegant mathematical formulations. These models offer the comforting illusion that adaptive behavior can be fully explained using familiar computational metaphors imported from engineering and artificial intelligence. However, this perceived elegance often comes at the expense of biological plausibility and scientific accuracy.

When we closely examine actual human movement data, the discrepancies between theoretical predictions and empirical observations become increasingly apparent (Loeb 2012, 2021). While such discrepancies rarely lead to formal rejection of internal models—given their theoretical flexibility, they expose how the framework often functions more as a post hoc convenience than an empirically constrained explanation. The models adapt, but only after the fact. In these cases, the search for internal models resembles what (Slaney and Maraun 2005, p.153) called an “analogy and metaphor running amok”—we risk projecting our own computational intuitions onto neural systems that may operate according to fundamentally different principles. This misalignment not only distorts our interpretations of neural and behavioral data but also narrows the space of viable alternative explanations grounded in the embodied dynamics of real biological systems.

The claim for internal models loses persuasiveness when it relies on ill-defined, theoretically vague, and methodologically unconstrained notions of representation (Chemero 2009; Hutto and Myin 2012). Without clearly specified, independently verifiable criteria and rigorously operationalized empirical tests, the internal model concept risks collapsing into a merely rhetorical construct rather than offering a scientifically explanatory framework (Keijzer 2001; Van Orden et al. 2001). This is not a hypothetical concern; internal model frameworks have already shaped experimental design, data interpretation, and theoretical commitments in ways that actively obscure alternative, more mechanistically grounded explanations. For example, studies of reaching under uncertainty routinely interpret variability as evidence of Bayesian priors and forward models without independently validating the representational mechanisms they assume (Fernandes et al. 2014; Körding and Wolpert 2004). This perspective offers a sustained and interdisciplinary critique of internal model principles within the domain of sensorimotor control by systematically examining their philosophical assumptions, methodological limitations, and persistent empirical contradictions. I argue that sensorimotor control is better understood as emergent, context-sensitive, and dynamically entangled with bodily and environmental constraints (Kelso 1995; Turvey 2007; Warren 2006)—not representational in any scientifically meaningful or testable sense. To continue invoking internal models in the absence of empirical validation is not simply a theoretical overreach—it reflects a systemic refusal to let data lead theory.

The conceptual ambiguity of the term *internal model* plays a central role in sustaining its dominance. The phrase is used across fields in at least three distinct ways: as a metaphorical heuristic, as a mechanistic claim about neural implementation, and as a mathematical formalism. These uses are rarely distinguished, allowing proponents to evade critique by shifting between meanings—deflecting philosophical challenges by retreating to metaphor, and claiming explanatory power by invoking mechanism. This semantic fluidity makes the theory difficult to falsify, intellectually incoherent, and methodologically irresponsible. It allows internal model frameworks to absorb counterevidence without consequence, not by resolving contradictions but by redefining what the theory is supposed to be (**Table** 1).

**Table 1:** A taxonomy of how the term *internal model* is used across fields. The ambiguity of usage contributes to the framework’s resilience despite empirical and conceptual problems

Usage type	Description	Problem/consequence
Internal model as metaphor	A heuristic borrowed from engineering to conceptually frame prediction and control; not intended to be literally instantiated in neural circuits	Evades empirical falsification; used rhetorically to defend theories when mechanistic claims fail
Internal model as mechanistic implementation	A literal claim that specific brain regions (e.g., cerebellum, motor cortex) instantiate computational representations of bodily dynamics	Lacks direct evidence; invites infinite regress and dualistic assumptions about control
Internal model as formal optimization device	A mathematical formalism (e.g., Bayesian estimators, Kalman filters) used to simulate behavior without commitment to neural realism	Conflates model fit with mechanism; easily reverse-engineered to match any behavior post hoc

The persistence of internal model thinking is not merely an academic inconvenience—it is an epistemological liability. By channeling empirical inquiry through the lens of preordained computational metaphors, researchers risk filtering out the very complexity that makes biological systems adaptive, resilient, and embodied. This is not just a theoretical misstep, but a systematic distortion of scientific practice: the internal model paradigm rewards models that resemble familiar artifacts—control systems, sensors, programs—regardless of whether they capture the actual operations of nervous systems. In doing so, it imposes explanatory closure on questions that remain wide open. The result is a body of literature that is algorithmically elaborate but biologically impoverished.

I have written this perspective for researchers across neuroscience, cognitive science, motor control, rehabilitation science, and robotics who engage—explicitly or implicitly—with internal model explanations. I speak equally to theorists and experimentalists: to those who build formal computational models of sensorimotor function, as well as to those who design behavioral experiments, interpret variability, or develop clinical and technological interventions. Philosophers of science and modeling will also find in this critique a detailed case study in how entrenched metaphors can eclipse mechanistic understanding. The present critique argues that internal model frameworks conflate engineering metaphors with biological explanation, thereby obscuring the embodied, dynamic, and context-sensitive nature of real-world sensorimotor behavior. Our goal is not to dismantle modeling per se, but to challenge the continued dominance of a specific, representationalist logic that has outlived its explanatory usefulness and empirical justification.

Critiques of internal, inferential models are not new. Foundational work by Gibson (1966, 1979), Bernstein (1996), and Turvey and colleagues (2014, 2018) have long challenged the idea that perception and action rely on internal representations, instead proposing alternative explanatory frameworks grounded in direct perception, motor redundancy, and principles of self-organization. More recent work by Chemero (2009), Barrett (2011), and others has extended and sharpened these critiques in response to contemporary debates in cognitive science and neuroscience. The present manuscript builds on these traditions but aims to make several distinct contributions: (1) to synthesize empirical and conceptual objections across neuroscience, psychology, biomechanics, and philosophy into a unified critique; (2) to integrate recent findings in multifractal and nonlinear movement analysis that pose fundamental challenges to internal model assumptions; (3) to expose the rhetorical, unfalsifiable, and circular logic that allows internal models to persist despite repeated empirical failures; and (4) to propose a biologically coherent alternative rooted in ecological dynamics, metastability, and distributed control. This work is not intended as a purely theoretical intervention, but as an interdisciplinary reckoning with the continued dominance of a framework whose explanatory power has been overstated and whose biological plausibility remains unproven.

Recent work by Raja (2024) offers a complementary lens for understanding the persistence and influence of internal model frameworks. Drawing on the concept of *motifs*, Raja characterizes the foundational assumptions of scientific programs as open-ended conceptual anchors—such as the motif of encoding or the motif of input–output systems—that shape explanation even in the absence of precise mechanistic commitments. From this perspective, internal models are not merely technical frameworks, but expressions of deeper theoretical motifs that structure inquiry within mainstream cognitive neuroscience. In contrast, radically embodied approaches—including ecological and dynamical systems theories—are guided by fundamentally different motifs, such as complex stimulation and resonance. While Raja’s aim is not to critique the mainstream framework, his analysis reinforces the idea that the dominance of internal models reflects a clash of foundational commitments rather than a settled empirical consensus. Identifying these motifs makes visible what is often implicit: that competing frameworks are not merely trading empirical claims, but operating from incommensurable conceptual ground. Framed in this light, the present manuscript can thus be read as a challenge not only to specific internal model implementations, but to the broader motif of inferential computation that continues to scaffold mainstream neuroscience despite mounting empirical and conceptual problems.

## Internal models in theory and practice

The challenges facing internal model theories may be less mathematical than conceptual: nature doesn’t compute with internal models, it moves through embodied, context-sensitive coordination where “good enough” isn’t a compromise, but the very logic of life.

### The definition and implicit assumptions of internal models

Internal model frameworks assert that the nervous system constructs and maintains neural representations of the body and environment in order to predict the sensory consequences of motor commands and to plan future actions (Shadmehr and Krakauer 2008; Wolpert and Kawato 1998). These frameworks elevate a computational metaphor to a mechanistic dogma: that the brain is a prediction machine solving abstract control problems through algorithmic simulation. Within this paradigm, the brain supposedly implements:

- **Forward models** to anticipate the sensory outcomes of motor signals based on efference copies—that is, internal duplicates of outgoing motor commands used for prediction
- **Inverse models** to compute the motor commands for needed to realize desired outcomes, effectively inverting the causal chain

This conceptual scheme is propped up by a litany of assumptions rarely examined and even more rarely justified:

- That the brain contains explicit, manipulable representations of bodily and environmental dynamics
- That such representations function like formal models with robust predictive capacity
- That sensorimotor challenges admit well-posed, tractable solutions
- That neural “circuits” operate like centralized control algorithms issuing commands to a passive body

These assumptions are not only biologically naive but also philosophically anachronistic. Not all challenges to internal model frameworks operate at the same level. Some reflect empirical contradictions—cases where observable data violate core model predictions (e.g., the structured, multifractal nature of variability). Others involve conceptual problems (e.g., the homunculus fallacy), or theoretical contrasts in which different frameworks interpret the same phenomena in fundamentally different ways (e.g., predictive computation vs. perception–action coupling). **Table** 2 clarifies these distinctions by contrasting internal model assumptions with critiques drawn from empirical findings, conceptual limitations, or alternative theories. These frameworks inherit the architecture of 20th-century control engineering and retrofit it onto a living, nonlinear, adaptive organism. But biological systems are opportunistic, distributed, and perpetually enmeshed in a context they do not control. Their dynamics are recursive, noisy, and co-regulated across scales. Internal model frameworks, by contrast, abstract away precisely what matters: embodiment, context, and time (Brooks 1991; Thompson and Varela 2001).

**Table 2:** Core assumptions of internal model frameworks and their empirical or conceptual challenges

Internal model assumption	Empirical/theoretical challenge
Neural circuits represent body/environment explicitly	Neural activity is context-sensitive and relational; stable representational mappings are difficult to isolate empirically
Movement is guided by predictive computation	Alternative theoretical frameworks propose that movement emerges through continuous perception–action coupling rather than forward simulation
Sensory prediction errors update internal models	Requires interpretive mechanisms (homunculus problem); empirical evidence for such internal comparison processes remains inconclusive
Brain is central controller issuing commands	Theoretical shift toward distributed and co-regulated control across neural, muscular, and biomechanical systems
Variability is noise around optimal trajectory	Empirical studies show structured, task-sensitive, and often functional variability (e.g., multifractality), inconsistent with standard noise models

### The engineering origins: from control theory to neural control

Internal model frameworks did not emerge from biology—it was imported from engineering. Specifically, from control theory, where it functions quite well in artificial systems designed with centralized architectures and explicitly coded control loops (Dupuy 2009; Wiener 1948). These systems embody a clean distinction between the controller and the controlled, operating with formalized mathematical models that mediate between desired goals and physical execution. But what works for thermostats and robotic arms falters when foisted onto living organisms. The direct transfer of engineering concepts to biology commits a category error so fundamental it ought to be disqualifying: it confuses human artifacts with evolved systems, projection with explanation (Rosen 1991; Pfeifer and Bongard 2006).

Living systems were not designed. They did not emerge from blueprints. They are the result of evolutionary bricolage—piecemeal adaptations shaped by the contingent forces of survival, development, and embodiment. There is no central control module, no omniscient system architect. Biological movement arises not from code executed by circuits but from recursive, co-regulated exchanges across neural, muscular, and environmental systems (Gibson 1979; Reed 1996). Treating such systems as if they instantiate engineering diagrams is not just misleading—it is inconsistent with biological principles. To claim that organisms “implement” control-theoretic architectures is to confuse the retrospective logic of design with the opportunistic chaos of evolution.

These mismatches become glaringly obvious when we examine real behavior. Take the case of grasping: in the engineering imagination, this is a multi-stage computation involving object localization, trajectory planning, and grip-force calibration—all supposedly governed by internal models (Flanagan and Wing 1997; Hwang et al. 2003; Kawato 1999; Todorov and Jordan 2002). But in the living body, grasping is not computed—it is enacted; the hand shapes itself in real time, informed by direct visuomotor coupling and affordances, not internal simulations (Smeets and Brenner 2008; van Doorn et al. 2007). The nervous system does not rehearse the grasp before acting; it participates in the act, adjusting dynamically to emergent conditions (Castiello and Begliomini 2008; Janssen and Scherberger 2015). Neural activity reflects this fluid integration, not the stepwise execution of a control algorithm, as, for instance, suggested by Kalaska et al. (1997). To portray grasping as an internal calculation is to mistake an improvisational dance for a spreadsheet. Such examples reveal not isolated misinterpretations, but a systemic misalignment between the assumptions of model-based control and the realities of situated action (**Table** 3).

**Table 3:** Examples of behaviors often attributed to internal models and their alternative explanations

**Behavior/phenomenon**	**Internal model explanation**	**Alternative explanation**
Reaching adaptation to force fields	Updating internal forward/inverse models	Dynamic reorganization of synergies
Cerebellar activation during movement	Predictive computation via forward model	Timing control, error correction, not representation
Cortical tuning to movement parameters	Encoding of inverse model parameters	Emergent attractor dynamics
Visuomotor coordination in grasping	Simulated internal reconstruction of object dynamics	Direct perception and online affordance use
Response variability in uncertain tasks	Bayesian priors and internal state estimation	Structured, task-driven variability

And yet, under the spell of engineering metaphors, researchers routinely impose computational narratives onto neural activity that may have no such structure. Every spike train becomes a representation; every motor output, a decoded signal. The metaphor becomes method, and the method becomes myth. This habit of interpretation not only distorts our understanding of brain function—it reifies a fiction. It leads us to attribute to the nervous system a model-building prowess it almost certainly does not possess.

While internal models may provide useful approximations in tightly constrained contexts (e.g., cerebellar oculomotor prediction), their generalization to embodied movement is a leap more rhetorical than empirical. What begins as a metaphor ends as dogma. In short, internal model frameworks reveal more about the engineers and theorists who constructed them than about the organisms they purport to explain. It is not a theory grounded in biological observation but one retrofitted from the logic of our own artifacts—our machines, our software, our metaphors. What began as a speculative analogy gradually hardened into methodological orthodoxy, calcifying into textbooks, grant proposals, and experimental paradigms. Along the way, it ceased to function as a guide for discovery and instead became a filter for interpretation. In doing so, it has not clarified the nature of biological control but has systematically obscured the very phenomena it set out to illuminate. Biology does not compute the world—it dwells in it, entangled with its environment through real-time, embodied action. Any theory that forgets this foundational truth risks explaining organisms out of existence, replacing life with a shadow cast by our own conceptual projections.

### The neuroscience of projection

Despite decades of dedicated research, the neural implementations of purported internal models remain stubbornly elusive (Ebner et al. 2011; Krakauer et al. 2017; Loeb 2012). While correlational evidence abounds—neural activity patterns that vary with task demands in ways consistent with model predictions—causal evidence that neural circuits actually compute and utilize representations in the manner proposed by internal model theories is notably lacking (Bechtel 1998; Haselager et al. 2003; Ramsey 2007). In many cases, the presence of task-related neural activity is interpreted as support for internal models simply because the model predicted something would happen, not because the neural activity unambiguously instantiates a representational mechanism. Alignment between model output and neural activity may suggest compatibility, but it does not confirm that the brain implements the model—it merely shows that the model can mimic behavior generated by very different mechanisms. Yet in a highly interconnected, multivariate system like the brain, nearly any neural signal can correlate with task-related behavior under some conditions—a fact that renders such correlations inevitable and, without independent constraints, scientifically inert. There is no parsimonious or principled way to attribute representational specificity to these patterns when the same system can flexibly correlate with many outcomes depending on context. This evidentiary ambiguity is especially apparent in claims about the cerebellum and motor cortex, which are routinely cast as the neural homes of forward and inverse models, respectively.

Research on cerebellar function exemplifies this evidentiary gap. The cerebellum has long been proposed as the neural substrate for forward models due to its anatomical organization and connectivity patterns (Ito 2008; Wolpert et al. 1998). Theoretical models elegantly map cerebellar circuitry onto the computational requirements of forward modeling, with parallel fibers representing context signals, Purkinje cells implementing predictive computations, and climbing fibers carrying error signals (Kawato 1999; Doya 1999). However, direct evidence that the neurophysiology of cerebellar circuits (Holdefer et al. 2000) actually implements these computations as proposed—rather than contributing to movement coordination through other mechanisms—remains inadequate. The appeal of these mappings reflects a deeper tendency to treat anatomical regularity as algorithmic intent—a move that stabilizes the internal model narrative without empirically validating it.

Similarly, the search for inverse models in motor cortical regions has yielded correlational patterns consistent with computational predictions but has failed to establish that these regions actually implement the proposed representational functions (Diedrichsen et al. 2007; Scott 2004). Neural activity patterns in primary motor cortex correlate with movement parameters and adapt to novel dynamics in ways broadly consistent with internal model predictions (Gandolfo et al. 2000; Gribble and Scott 2002; Shadmehr and Krakauer 2008). However, alternative interpretations of the same data suggest that these patterns might reflect dynamic engagement with task constraints rather than the computational implementation of internal models (Churchland et al. 2012; Shenoy et al. 2013). In both cerebellum and cortex, the same empirical pattern—task-related modulation—is treated as evidence of computational implementation. Crucially, these neural responses are equally compatible with non-computational accounts based on sensorimotor coupling, system dynamics, or task-level affordances (Gibson 1979; Noë 2004; Warren 2006)—none of which require internal modeling to explain adaptive behavior.

Taken together, these empirical and theoretical shortcomings raise a troubling possibility: perhaps we are not discovering internal models in the brain, but rather imposing them—retroactively and rhetorically—onto systems that operate according to fundamentally different principles. This possibility gains further credence when we consider that internal model frameworks emerge from engineering disciplines (Kalman 1960) rather than from direct observation of neural function. We may be asking the wrong questions, chasing the wrong signals, and mistaking structural regularity for algorithmic intent—not because the data compel such interpretations, but because the framework itself filters observation through a computational lens it cannot critically examine (Barrett 2011; Chemero 2009).

This confusion between correlation and attribution is pervasive in internal model research. Neural signals that co-vary with task variables are frequently taken as evidence that the brain is *computing* internal models, when in fact such signals may simply reflect dynamic covariation with behavior, sensory inputs, or biomechanical context. Yet internal model claims often elude falsifiability, violating even minimal Popperian standards for what constitutes a meaningful and testable scientific hypothesis (Popper 2002). In systems as richly interconnected and context-sensitive as the brain, such covariation is expected—and without independent constraints, attributing it to a specific internal computation amounts to little more than theoretical projection. This is not an argument against modeling per se—modeling is essential to science—but against the intellectual imperialism of a single, engineering-inspired architecture that has been elevated from a heuristic into a universal explanatory framework. True attribution would require interventionist or falsifiable evidence showing that removing or modifying the signal impairs behavior *specifically* in the way predicted by internal model theory. This bar is rarely met.

A recurring error in internal model research is the inference from empirical correlation to mechanistic instantiation. The fact that neural activity patterns co-vary with behavioral variables in ways consistent with a model’s predictions does not establish that the brain is implementing that model. Correlation, even when statistically robust, is not evidence of causal mechanism (Glymour 2001; Pearl 2009). This distinction is especially critical when model parameters are tuned post hoc to fit the data, creating a superficial alignment that may reflect the model’s flexibility rather than the system’s architecture. In such cases, what appears to be empirical confirmation may instead be an artifact of calibration. Treating this alignment as causal evidence collapses explanation into mimicry.

What we are witnessing is not scientific progress but methodological performances that may obscure underlying conceptual issues—elaborate performances of computational rigor that mask the emptiness at the core. Laboratories continue to receive millions in funding to map neural “implementations” of models that have never been shown to exist, while alternative frameworks languish for lack of computational glamor. This represents not just poor science, but significant opportunity costs in terms of research directions and resource allocation.

## The philosophical and logical problems with internal models

Imposing representational frameworks on neural systems isn’t just a technical mistake—it’s conceptual violence, forcing living, context-bound processes into the straightjacket of our own computational delusions.

### The homunculus problem: who interprets the representation?

**Fig. 2:**
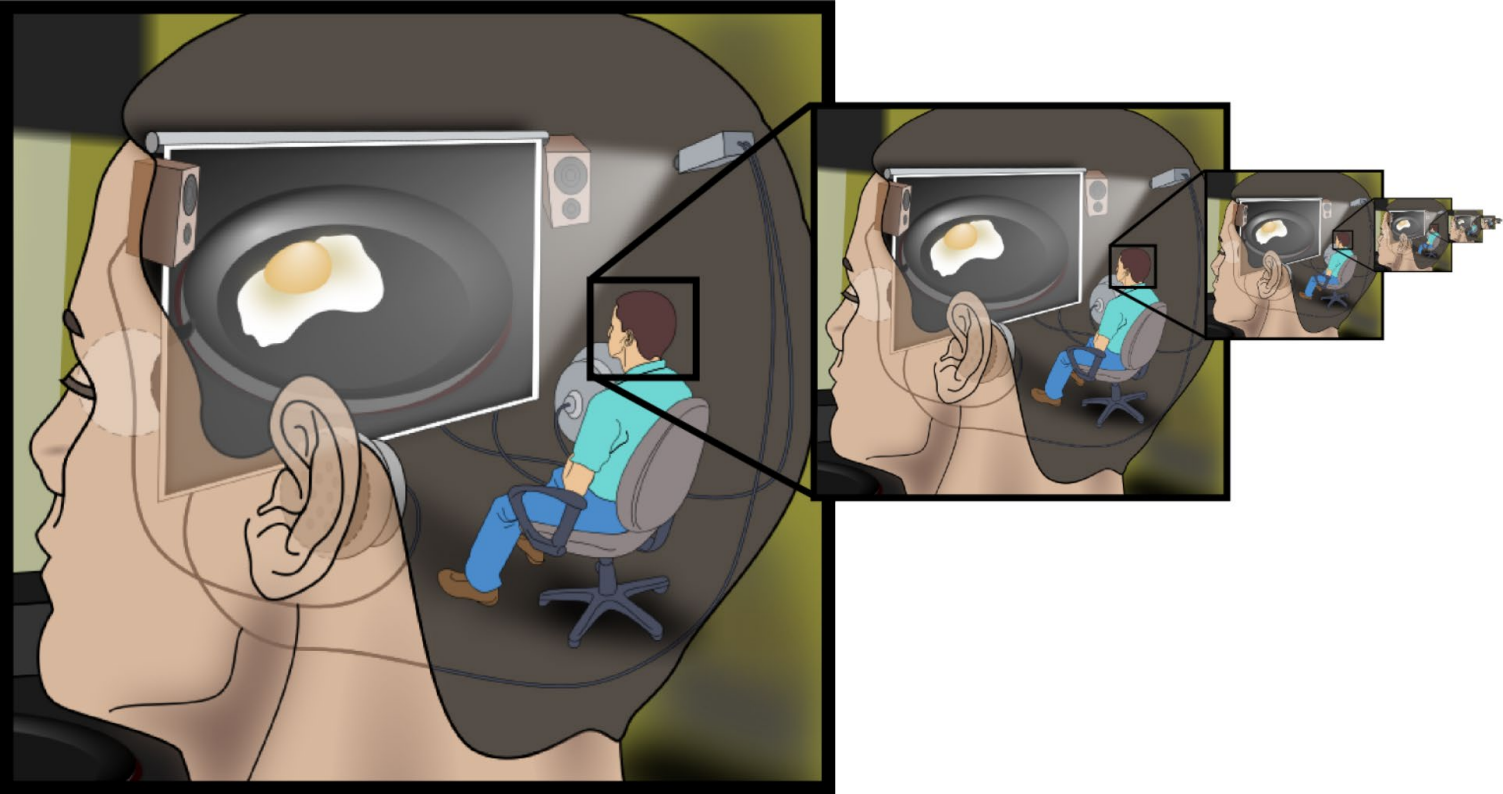
The homunculus problem in internal model theories. Internal model frameworks implicitly presume an internal agent—an interpreter within the nervous system—that selects, compares, and adjusts predicted outcomes based on pre-specified criteria. The recursive imagery of a person with a smaller person inside their head watching a monitor—each one containing yet another—illustrates the infinite regress this assumption entails. At its core, the framework projects intentionality into neural tissue by presupposing that some subcomponent of the system “knows” what to compare, without ever explaining how such agency arises. This logical paradox reveals how internal model theories smuggle decision-making into mechanistic accounts under the guise of computation, substituting metaphor for mechanism. What appears to be an elegant model of sensorimotor control is, upon closer scrutiny, a circular narrative anchored in conceptual convenience rather than biological reality. (Image credit: Jennifer Garcia, CC BY-SA 2.5.)

One of the most conceptually corrosive features of internal model theories is the homunculus problem—a hidden illusion smuggled in under the guise of mechanistic rigor and computational precision (Dennett 2017; Thompson and Varela 2001). When we claim that the nervous system utilizes a forward model to predict sensory consequences, we must ask: Who or what within the system interprets this prediction, assesses its relevance, and decides what to do with it? If the prediction itself is a neural representation, it requires interpretation to be functionally useful—which in turn demands another interpreter, and then another—triggering an infinite regress of nested homunculi (Fig. 2; Ryle and Tanney 2009; Thompson 2010).

Internal model theories often attempt to sidestep the regress by appealing to computational mechanisms that automatically process representations without requiring conscious interpretation or internal deliberation (Ito 2008; Kawato 1999; Schaal and Schweighofer 2005; Shadmehr and Krakauer 2008; Wolpert et al. 1998; Wolpert and Ghahramani 2000). However, this maneuver merely pushes the homunculus problem down a level without resolving its underlying logic. Delegating interpretation to an unconscious subroutine does not dissolve the need for interpretation—it only obscures it. Even so-called automatic computational mechanisms that utilize internal representations must still implement a means of extracting functional meaning from those representations in order to generate context-appropriate outputs (Bechtel 1998; Haselager et al. 2003; Ramsey 2007). Without an account of how this semantic extraction occurs, the theory reintroduces agency under the hood while pretending to eliminate it.

Consider a forward model predicting the sensory consequences of a reaching movement. What appears to be a clean computational sequence is, in fact, a carefully staged theoretical performance—one that quietly reintroduces interpretive agency at every step. For this prediction to be functionally useful in motor control, the system must:

- Generate the prediction based on an efference copy
- Compare the prediction with actual sensory feedback
- Calculate the discrepancy between predicted and actual outcomes
- Use this discrepancy to update the model and adjust motor commands

What these frameworks present as a mechanistic explanation is, in fact, a semantic sleight of hand—an elaborate substitution of process for principle, where meaning is presumed but never grounded. Each of these steps conceals the same core fallacy: that interpretation, evaluation, and correction happen without an agent to interpret, evaluate, or correct. Internal models do not explain agency—they reproduce it in miniature, again and again (Bennett and Hacker 2022; Hutto and Myin 2012). At no point does the framework explain how these computational steps become meaningful within the system, nor does it specify the biological mechanisms by which representations are extracted, compared, or revised. The result is a description of control that remains functionally dependent on implicit decisions and internal comparisons—none of which have been empirically observed in the brain. This logical problem is not merely a philosophical curiosity—it undermines the explanatory coherence of the entire internal model framework by introducing circular reasoning at its conceptual core (Table 4).

**Table 4:** Major philosophical problems associated with internal model frameworks

Philosophical problem	Description	Implication
Homunculus problem	Requires an interpreter to use internal predictions	Leads to infinite regress
Symbol grounding problem	No explanation for how neural symbols acquire meaning	Representational claims remain empty
Cartesian theater	Separates controller and controlled (brain vs. body)	Reinstates mind–body dualism
Circularity	Behavioral models infer representations, then use behavior to confirm them	Invalidates independent testing
Unfalsifiability	Any empirical data can be accommodated post hoc	Violates criteria of scientific theory

### The symbol grounding problem in sensorimotor control

Closely related to the homunculus problem is the symbol grounding problem—the question of how neural representations acquire their meaning or reference in the first place (Bickhard 2009; Harnad 1990). In the context of sensorimotor control, this becomes the challenge of explaining how specific patterns of neural activity come to represent bodily states, environmental properties, or causal dynamics of the world (Chemero 2009; Ramsey 2007). That is, what makes a spike train in the motor cortex mean “elbow angle” or a pattern of cerebellar activation mean “impending sensory error”?

Internal model theories sidestep this foundational question by assuming that neural signals correspond to meaningful features of the world—that a representation “just is” their target. But this assumption smuggles in the very thing it needs to explain: the mapping between internal signals and external referents (Hutto and Myin 2012; Ramsey 2007). They treat semantic content as intrinsic to neural activity rather than as something that must be constituted through relational engagement with the environment. Without an account of how this mapping is established, sustained, and functionally grounded within a system that lacks an interpretive observer, the framework leaves a conceptual hole in place of a mechanism. It replaces explanation with correlation and assumes meaning where none has been shown to emerge.

This problem becomes especially acute in the domain of sensorimotor control, where representations—if they exist—would need to track rapidly changing, high-dimensional bodily states and environmental affordances in real time. For a neural activity pattern to “stand in” for a moving limb or an external obstacle, the mapping between neural activity and the represented content must be dynamically updated and stably decoded under constantly shifting conditions (Smith and Thelen 2003; Turvey 2007). But internal model frameworks rarely explain how such mappings are formed, how they remain stable across movement contexts, how they generalize to new contexts, or how they remain meaningful to the system without invoking a hidden interpreter. And without this stability, the entire architecture collapses: a representation that loses its referent mid-action is not a control mechanism—it is a liability. The result is a representational vocabulary that sounds explanatory but fails to account for the most basic requirement of a representational system: that its internal signals are not just correlated with, but grounded in, the things they supposedly represent (Bechtel 1998; Haselager et al. 2003; Ramsey 2007).

### The Cartesian theater revisited

Despite presenting themselves as mechanistic accounts of neural function, internal model theories implicitly resurrect a form of Cartesian dualism—what Dennett aptly termed “the Cartesian theater”—by treating the brain as a detached control center that observes, simulates, and directs the body from a representational command post (Dennett 1991). Rather than dissolving the mind-body split, these frameworks quietly reintroduce it through computational metaphors that separate the locus of control (the brain) from the physical substrate being controlled (the body). In doing so, they repackage the ghost in the machine as a centralized algorithm issuing commands to a peripheral actuator (Chemero 2009; Thompson and Varela 2001). What looks like explanation is just choreography for a theater that never existed. This dualistic architecture becomes most apparent in the tripartite structure of internal model frameworks:

- The controller: a neural system containing an internal representation of the body and environment
- The controlled: the passive, mechanical body awaiting instruction
- The representation: an internal simulation that allegedly mediates between the two

This split-stage formulation installs a cognitive puppet master (the controller), a compliant marionette (the body), and an omniscient screen (the representation)—a fable of centralized control superimposed onto a system built through distributed, embodied interaction (Thelen 1995; Thompson 2010). It is not an explanation of life—it is a script written for a robot and imposed on an organism that was never engineered. Neural activity does not merely “represent” bodily states—it participates in their ongoing construction through dynamic, recursive coupling with musculoskeletal and environmental constraints (Clark 1998; Thompson and Varela 2001).

The Cartesian theater problem becomes especially conspicuous in discussions of prediction error, where internal model theories claim that discrepancies between predicted and actual sensory outcomes generate “error signals” that drive model revision and behavioral adaptation (Shadmehr and Krakauer 2008; Wolpert and Kawato 1998). But this framing presupposes an internal spectator—some evaluative subsystem that monitors the prediction, detects the mismatch, and selects the appropriate correction. It replaces a body moving through the world with a brain watching a movie of itself.

This framework does not merely obscure embodiment—it erases it, replacing lived coordination with abstract control logic scripted in advance and decoupled from the real-time contingencies that define biological action (Clark 1998; Thompson and Varela 2001). Biological movement is not a sequence of discrete computations—it is a temporally extended, improvisational process that emerges from the interplay of neural, bodily, and environmental dynamics. The insistence on carving it into modular components for representation, comparison, and command is not just conceptually misguided—it is ontologically incoherent, rooted in a view of the organism as a machine rather than as a self-organizing, embodied system (Thelen and Smith 1994; Turvey 2007) (Tables 5, 6).

**Table 5:** Empirical criteria for representational internal models versus current empirical evidence

Required criterion	Status in current research
Direct observation of neural representations	Not achieved
Causal disruption of specific models impairs behavior	Rare, and when found, often has multiple interpretations
Falsifiable predictions derived from model	Most predictions are post hoc or fit-after-the-fact
Consistent mapping from neural activity to model use	Neural activity is flexible, task-dependent, and multivariate
Converging evidence across levels of analysis	Mostly behavioral; neural evidence is ambiguous or indirect

**Table 6:** Comparison of internal models with alternative frameworks in sensorimotor control

Framework	Control mechanism	Role of representation	Adaptation mechanism	Variability interpreted as
Internal models	Central computation of predicted outcomes	Essential	Model updating through prediction errors	Noise around optimal trajectory
Ecological psychology	Direct perception of affordances	Unnecessary	Perception–action recalibration	Functional variability
Dynamical systems	Self-organization and attractor dynamics	Not required	Parameter-driven phase transitions	Structure supporting flexibility
Synergetics	Soft-assembled coordinative structures	Not invoked	Degeneracy and uncontrolled manifolds	Functional component of coordination
Non-representational prediction	Metastable sensorimotor contingencies	Implied through dynamic coupling	System attunement through practice	Indicator of anticipatory flexibility

## Evidence against internal models in biological systems

After decades of fruitless searching, the lack of neural evidence for internal models isn’t just a gap—it’s a theoretical collapse. We may have been chasing a computational chimera: a tidy engineering fantasy with no basis in the messy reality of embodied, ecological movement.

### The failure to localize representational functions

Despite decades of intensive research, neuroscience has consistently failed to identify clear and convincing neural implementations of internal models—hypothetical forward and inverse representations long presumed to underlie voluntary motor control. While neural activity often correlates with task variables in ways that conveniently align with model predictions, causal evidence that these brain regions actually compute, store, or utilize such internal models remains conspicuously and persistently absent. This empirical vacuum spans both forward and inverse models, raising serious doubts about whether these constructs reflect biological mechanisms at all—or whether they are theoretical mirages, projections of our engineering intuitions onto neural tissue.

#### The cerebellar forward model hypothesis

The cerebellum is often cast as the brain’s predictive engine, allegedly implementing forward models that anticipate sensory consequences of motor commands (Ito 2008; Wolpert et al. 1998). Yet this narrative disintegrates under scrutiny:

- **Causal disruptions are ambiguous:** Cerebellar damage impairs movement coordination, but these impairments are not specific to prediction failure and can arise from deficits in timing, feedback integration, or sensorimotor association (Bastian 2006; Diedrichsen et al. 2005).
- **Neural activity is non-specific:** Cerebellar neurons exhibit patterns that reflect both incoming sensory and outgoing motor signals, making it impossible to isolate a predictive computational role (Ebner et al. 2011; Lisberger 2009).
- **Simpler explanations suffice:** The cerebellum’s observed functions—such as error correction, temporal coordination, and associative learning—account for its activity without requiring the machinery of internal models (Ivry and Spencer 2004; Manto et al. 2012).

In particular, the timing hypothesis offers a biologically grounded alternative: cerebellar circuits may serve to align sensorimotor events in time rather than simulate the future (Ivry and Spencer 2004; Mauk and Buonomano 2004; Spencer and Ivry 2009). This view explains cerebellar activation during “predictive” tasks not as model-based foresight, but as the coordination of temporal structure in embodied action.

#### The cortical inverse model hypothesis

Claims that the motor cortex implements inverse models—mapping desired movements to the necessary motor commands—fare no better. The evidence remains overwhelmingly correlational and deeply ambiguous:

- **Population dynamics defy computation:** Motor cortical activity unfolds as high-dimensional, time-evolving trajectories rather than discrete encodings of desired kinematic states (Churchland et al. 2012; Shenoy et al. 2013).
- **Neural responses are multiplexed:** Individual neurons respond to multiple movement parameters at once (Fetz 2007; Scott 2003), making it nearly impossible to isolate any singular “inverse” computation.
- **Better models exist:** Dynamical systems frameworks interpret cortical activity as trajectories evolving in state space, coordinating with peripheral dynamics. This perspective offers an alternative to representational accounts, though the cited work characterizes such trajectories within cortical populations (Churchland et al. 2012; Kaufman et al. 2014).

The inverse model hypothesis, though computationally convenient, fails to capture the distributed, context-sensitive, and time-dependent nature of cortical activity observed in real biological systems. This dynamical view is not just theoretically elegant—it is empirically grounded. It treats the motor cortex not as a calculator of desired outcomes but as a pattern generator tightly coupled to the body’s biomechanics and environmental context. This perspective reframes the cortex from a representational problem-solver to a participant in a distributed, real-time control architecture—one that does not need to “model” the world to move effectively within it.

### The problem of coordinated complexity

Beyond the failure to localize internal models, these theories face a deeper challenge: they cannot account for how the nervous system coordinates the vast complexity of movement without collapsing under the weight of a combinatorial explosion (Huys et al. 2003; Turvey 2007). The human body contains over 600 muscles and 200+ degrees of freedom in the joint space, creating a control problem of astronomical dimensionality if tackled through explicit computation (Bernstein 1996; Turvey 1990). To salvage tractability, internal model theories rely on simplifying assumptions—modular decomposition, dimensionality reduction, and optimal control heuristics—that shrink the problem space into something solvable (Flash and Hogan 1985; Todorov 2005). But these shortcuts introduce new theoretical liabilities:

- **Modularity fails under real-world variability:** Biological movement is exquisitely context-sensitive, yet modular control schemes rarely explain how coordination patterns adapt fluidly to novel or shifting demands without incurring switching delays or coordination breakdowns (Feldman 2009; Latash 2008).
- **Dimensionality reduction begs the question:** These approaches require pre-selecting “relevant” dimensions—but how does the nervous system determine relevance without another layer of computational overhead? The solution smuggles in the very complexity it aims to eliminate (Latash 2008; Tresch and Jarc 2009).
- **Optimal control creates circular logic:** These models reverse-engineer objective functions from observed behaviors, then claim to explain those behaviors as outcomes of optimization—essentially predicting the data from the data (Loeb 2012; Valero-Cuevas et al. 2009).

What emerges is a fundamental mismatch: while internal model theories need drastic simplification to survive, biological systems exhibit *coordinated complexity* as a native feature, not a computational burden (Kelso 1995; Turvey 2007). The seamless integration of movement, perception, and context suggests that coordination is not centrally computed but emergent—arising from the intrinsic dynamics of neural, biomechanical, and ecological systems working in concert (Thelen and Smith 1994; Turvey 1990).

**Fig. 3:**
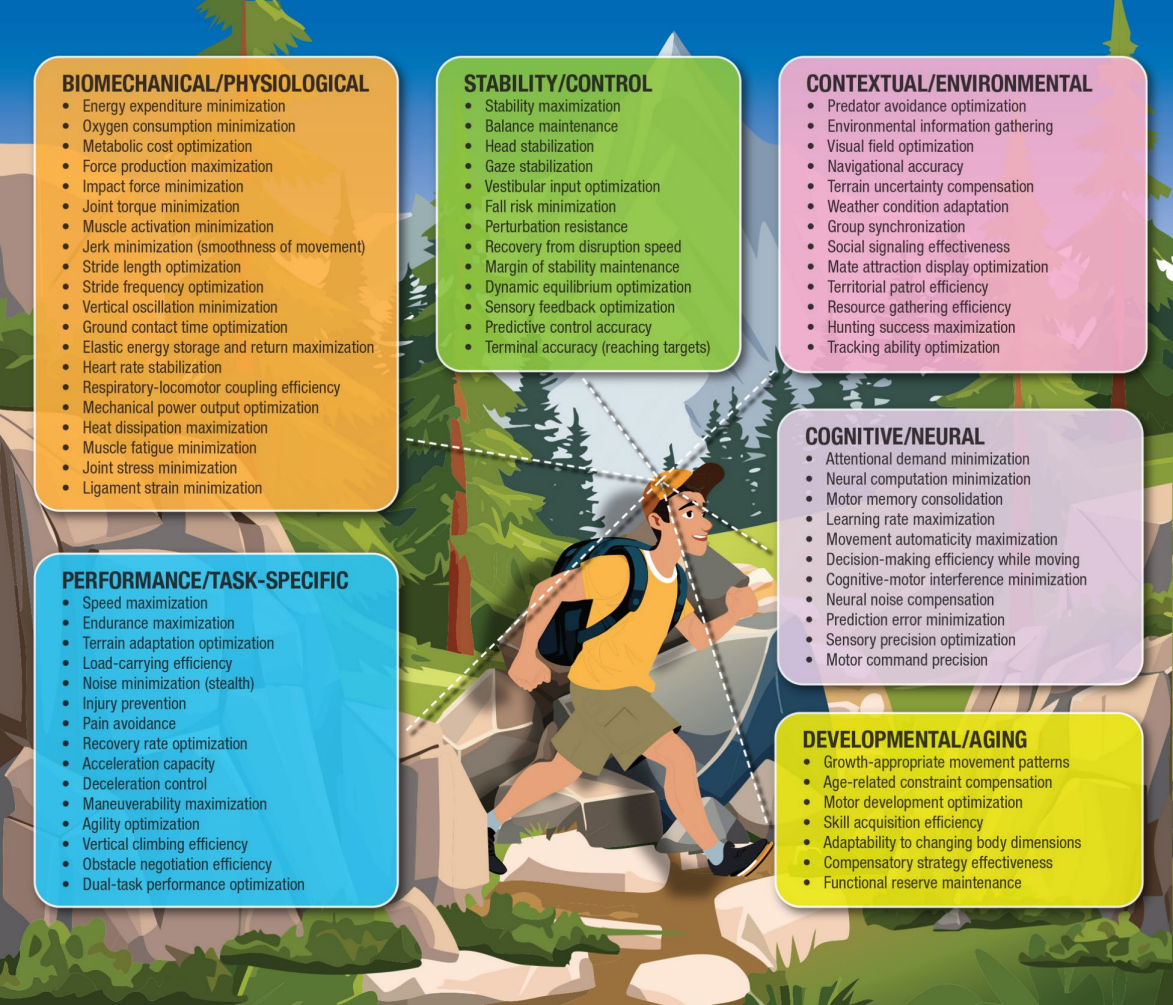
The multidimensional constraint space of human locomotion. Coordinated walking must simultaneously navigate constraints across biomechanics, stability, performance, neural control, environmental demands, and lifespan development. The combinatorial burden of representing and optimizing across this space is computationally intractable. This complexity challenges internal model frameworks, which presume that the nervous system can simulate or optimize across all these domains. A more plausible explanation lies in distributed, context-sensitive, and self-organizing coordination

If internal models compute sensorimotor behavior, what are they computing? Figure 3 illustrates the absurdity of the claim: there is no single solution to movement. Instead, the body operates within a high-dimensional constraint landscape that defies reduction to an internal simulation.

Rather than modeling the world, biological systems appear to exploit their own morphology, temporal dynamics, and environmental affordances to *embody* control—rendering many internal model assumptions not only biologically implausible, but conceptually obsolete.

### The context problem: task-specific adaptation without models

A further blow to internal model theories comes from the problem of context. Across domains, biological movement displays astonishing sensitivity to environmental conditions—with no evidence that such adaptation relies on explicit internal representations (Kelso 1995; Riley and Turvey 2002). Instead of updating models, the system appears to adapt directly, fluidly, and in real time. This context dependence is evident in a wide range of behaviors:

- **Postural control** continuously adjusts to surface properties, visual input, and task demands—without signs of internal model recalibration (Carver et al. 2017; Riley and Turvey 2002).
- **Manual coordination** reorganizes effortlessly in response to tool shape, spatial layout, and action goals, guided by direct perception-action coupling rather than representational planning (Smeets and Brenner 2008; van Doorn et al. 2007).
- **Locomotion** adapts seamlessly to changes in terrain, speed, and obstacles through emergent patterns of coordination—not pre-computed trajectories or look-up-table solutions (Patla 2004; Warren 2006).

To explain this flexibility, internal model theories would require an impractically vast library of pre-specified models or a computational system constantly updating high-dimensional predictive maps. Either scenario imposes a cognitive burden far beyond what biological systems plausibly carry (Feldman 2009; Raja et al. 2023; Loeb 2012; Valero-Cuevas et al. 2009).

The context problem exposes a deeper flaw: the assumption that adaptation must be mediated by internal representations. In reality, biological systems exhibit context-specific adaptation not through computation, but through attunement—emergent coordination with the environment rooted in morphology, dynamics, and ecological constraints (Gibson 1979; Kelso 1995). Movement is not computed and then deployed—it is lived in real time, shaped by the organism’s moment-to-moment entanglement with its environment.

### The development problem: learning without internal models

Another critical failure of internal model theories is their inability to account for the developmental emergence of sensorimotor coordination. From infancy through adolescence, sensorimotor skill is not pre-loaded, but incrementally assembled through active, embodied exploration. Models do not grow up—but people do. The transformations that define development–postural change, growth, locomotor transitions, and perceptual attunement—unfold not through the refinement of pre-specified representations, but through dynamic reorganization in response to bodily and environmental change. This reality directly contradicts the logic of internal models, which presume stable mappings between motor commands and sensory consequences. Developmental research provides extensive empirical evidence for non-representational mechanisms of learning:

- **Infant locomotion** evolves through continuous adaptation to changing body dimensions and support surfaces, not through generalized internal models (Adolph 2000; Adolph and Avolio 2000; Kretch et al. 2014).
- **Postural and slope negotiation** is re-learned after every developmental milestone (e.g., from crawling to walking), indicating absence of transfer across similar tasks with different morphologies (Adolph et al. 1993, 2010; Adolph and Tamis-LeMonda 2014; Cole and Adolph 2023).
- **Affordance perception** is acquired through real-time exploration, as shown by Eleanor Gibson’s work on perceptual learning, without reliance on abstract world models (Gibson and Pick 2000).

To explain these phenomena, internal model frameworks would require a combinatorially explosive architecture that recalibrates its internal representations with each change in body size, posture, or motor repertoire. No such system has been observed. Worse, the core assumptions of internal model theory—generalizability, prediction, and optimization—are directly contradicted by the task-specific, morphology-dependent learning trajectories observed in early development.

The development problem reveals a deeper and more consequential conceptual failure: learning is not the calibration of representations, but the discovery and stabilization of coordinative solutions through recursive organism–environment interaction. Sensorimotor skill is not a product of abstract inference, but of embodied attunement over time. Internal models are structurally and philosophically incapable of capturing this process. In contrast, ecological and dynamical systems frameworks place developmental transformation at the center of explanation, treating variability as functional and coordination as emergent and distributed. If internal models cannot explain how action becomes possible in the first place, their usefulness as accounts of control is fundamentally and irrevocably undermined.

### Multifractality and the limits of computational models

A growing body of evidence reveals that the variability in biological movement is not random noise but rather a structured, functional property of adaptive systems. Such complexity directly contradicts the assumptions of internal model theories, which typically rely on linear, time-invariant dynamics and treat variability as noise to be minimized or filtered out. Perhaps the most profound empirical challenge to internal model theories comes from the observation that human and animal movement exhibit multifractality—a mathematical signature of nested, nonlinear interactions that span across multiple spatial and temporal scales (Ihlen 2012; Kelty-Stephen et al. 2023). Multifractality describes how fluctuations at different timescales are not isolated or independent (as assumed in many models), but dynamically interdependent, forming a cascade of interactions in which smaller fluctuations modulate, and are modulated by, larger ones. This behavior is fundamentally multiscale, nonlinear, and non-Gaussian, in that it cannot be captured by simple averages or standard deviations, and the relationships between system components are not additive but multiplicative and context-sensitive.

To illustrate, consider a person standing quietly on a force plate. Rather than exhibiting small, random postural sways around a fixed equilibrium point—as predicted by linear control models or internal model frameworks—their center-of-pressure signal reveals a complex, temporally layered web of variability that extends from rapid millisecond-scale fluctuations to slower modulations over minutes. This variability is not reducible to random error or sensorimotor noise; rather, it exhibits multifractal structure—a hallmark of interaction-dominant dynamics, in which behavior emerges from the coordination of many interdependent elements operating across multiple scales of time and space (Riley and Turvey 2002; Kelty-Stephen et al. 2021; Kelty-Stephen and Mangalam 2022).

Multifractal analysis has revealed several key empirical findings that are incompatible with the assumptions of internal model theories:

- **Scale-free and long-range dependencies:** Movement variability shows power-law correlations across timescales. For instance, gait stride intervals, bodily fluctuations during haptic perception of handheld object properties, eye movements during visual search, and fluctuations in hand position during reaching tasks all exhibit long-range, scale-free structure (Carver et al. 2017; Ihlen and Vereijken 2013; Kelty-Stephen and Mirman 2013; Mangalam et al. 2020, 2020a, b; Mangalam and Kelty-Stephen 2020; Mangalam et al. 2021, 2023; Pratviel et al. 2021; Wilson et al. 2023). This stands in direct contrast to internal models that treat deviations as temporally uncorrelated noise around a target trajectory.
- **Interaction-dominant control:** Rather than reflecting independent components or modules operating in parallel, movement appears to emerge from continuous, nonlinear coupling across levels of the system. For instance, finger-tapping experiments show that fluctuations in inter-tap intervals are influenced not just by local motor processes but by interactions spanning cognitive, perceptual, and biomechanical systems (Ihlen and Vereijken 2013; Van Orden et al. 2003).
- **Multiplicative, cascade-like dynamics:** Multifractal analysis reveals that variability in biological movement is not merely the result of additive noise, but instead reflects multiplicative interactions that cascade across scales. These cascades are evident in both postural control and suprapostural tasks, where fluctuations at one timescale modulate, and are modulated by, fluctuations at other scales. For example, visual effort during quiet stance modulates cascade dynamics in postural sway (Mangalam et al. 2021), and multifractal nonlinearity in knee torque predicts adaptive responses to mechanical perturbations during a single-leg squat task, particularly under dual-task demands and aging-related declines in dexterity (Kelty-Stephen et al. 2023). Such findings are fundamentally incompatible with internal model frameworks that assume linear, time-invariant control structures and treat variability as a computational flaw rather than a functional resource.

The implications of these findings are profound. Internal model theories assume that movement is guided by stable internal representations that generate predictions and correct for errors through feedback. Deviations from expected trajectories are treated as noise—statistical anomalies to be minimized through optimization or estimation processes such as Kalman filtering or Bayesian inference (Ito 2008; Kawato 1999; Schaal and Schweighofer 2005; Shadmehr and Krakauer 2008; Wolpert et al. 1998; Wolpert and Ghahramani 2000). But this view collapses under the empirical weight of multifractality. In multifractal systems, variability is not noise—it is structure. It reflects the system’s capacity to adapt, to flexibly reconfigure across changing contexts and constraints. For example, in locomotion, stride-to-stride variability is not a sign of poor control but a signature of a healthy, exploratory system capable of adjusting to environmental perturbations, such as changes in terrain or sudden shifts in body posture. Moreover, the non-Gaussian nature of multifractal dynamics means that average-based models (which assume normal distributions of error) systematically misrepresent the true nature of biological movement. Large deviations, instead of being outliers, may be integral to the system’s dynamics—allowing for transitions, innovations, or corrective strategies that would be impossible under rigid predictive control.

**Fig. 4:**
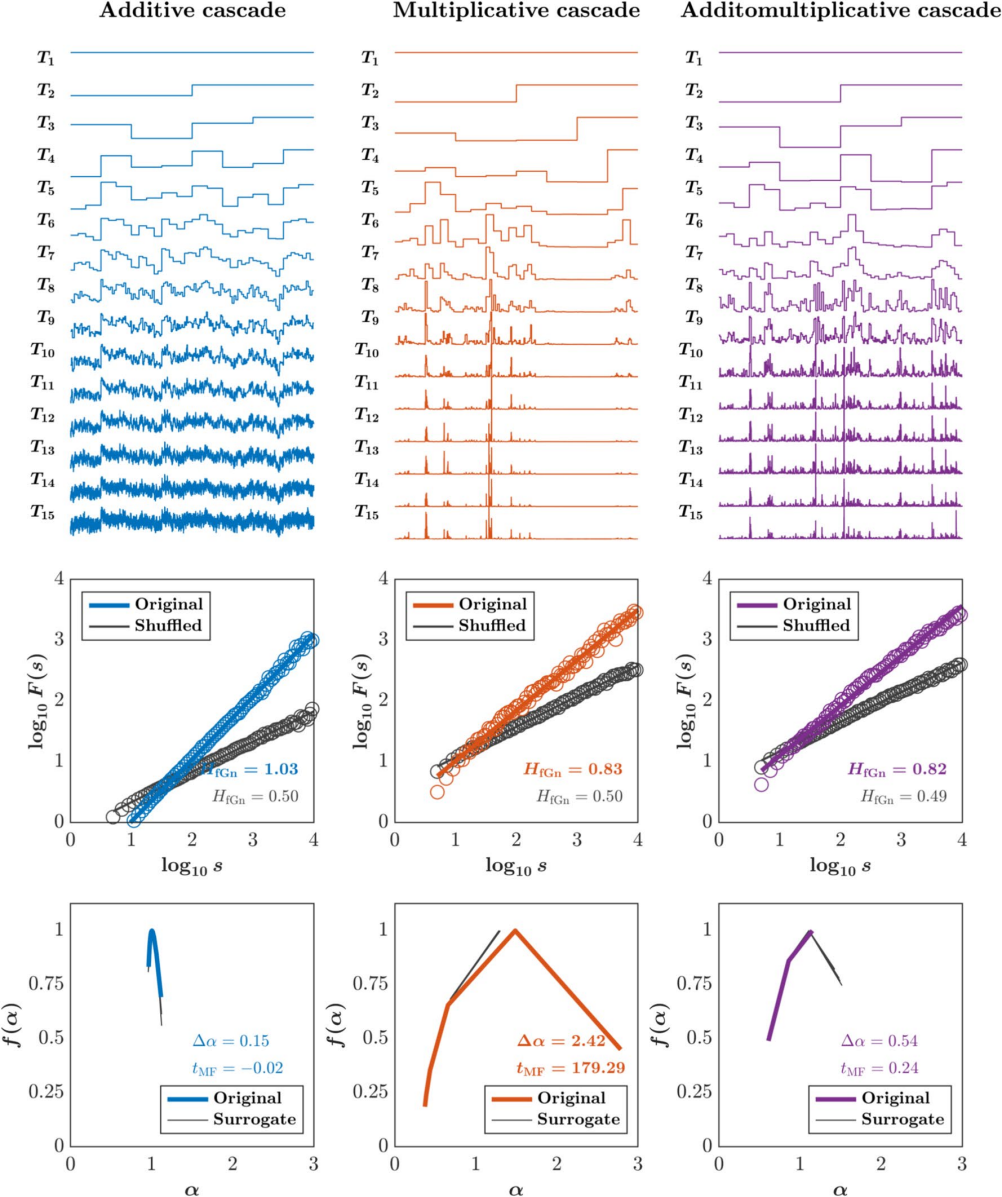
Multifractality is more sensitive to the structure of scale-dependent interactions than to monofractal scaling. Cascades were generated by progressively subdividing time intervals and injecting noise in additive (*left*), multiplicative (*center*), or hybrid additomultiplicative (*right*) fashion over 15 generations ($T_{1}$ through $T_{15}$). Although all cascades yield similar monofractal Hurst exponents ($H_{\text {fGn}} \; \approx \; 0.8$–1.0)—a measure of long-range temporal persistence, they differ substantially in multifractal spectrum width ($\Delta \alpha$) and nonlinear surrogate divergence ($t_{\textrm{MF}}$). This demonstrates that structurally different interactions—despite producing similar monofractal summaries—yield distinct temporal organizations only visible through multifractal and nonlinear analyses. Such distinctions underscore the limitations of internal model frameworks and monofractal approaches, which reduce structured variability to noise and miss the complexity of real-world sensorimotor dynamics. Reproduced from Kelty-Stephen and Mangalam (2024)

Figure 4 illustrates how the structure of interactions across scales—additive, multiplicative, or hybrid—shapes the emergence of multifractal and nonlinear dynamics. Although these cascades yield similar monofractal estimates of the Hurst exponent, their multifractal spectra and surrogate-based nonlinear signatures differ markedly. This divergence underscores a key argument of the manuscript: that interaction-dominant dynamics produce structured variability that internal model frameworks fundamentally mischaracterize as noise. Such distinctions are invisible to models that rely on averaging or assume modular, feedforward computation. By contrast, multifractal analysis reveals the nested, scale-sensitive complexity essential to understanding real-world sensorimotor coordination.

The multifractal perspective redefines variability—from an undesirable byproduct of imperfect computation to a functional hallmark of systems capable of resilience, flexibility, and learning. Multifractality reveals a deeper layer of biological intelligence—one that operates not through precision and control, but through openness, adaptability, and nested coordination across scales. Such intelligence is not localized in a central processor but distributed across the organism—environment system, unfolding dynamically through continuous interaction.

In sum, accommodating the empirical realities of multifractal dynamics would require internal model frameworks to incorporate increasingly complex, flexible, or post hoc assumptions—thereby weakening their explanatory parsimony and reinforcing concerns about their unfalsifiability. They lack the mathematical architecture to represent interaction-dominant systems, the conceptual flexibility to treat variability as functional, and the philosophical stance to see control not as the suppression of noise, but as the orchestration of structured fluctuation. Its assumptions about modularity, linearity, and central control stand in direct conflict with the multiscale, nonlinear, and non-Gaussian dynamics observed in real-world motor behavior.

While the presence of multifractal structure in postural sway, locomotor patterns, and exploratory movement underscores the inadequacy of linear control frameworks, its role within perception—action systems remains an open and unresolved question. The identification of multifractality in behavior does not, by itself, demonstrate that multifractal dynamics are directly perceived, nor that they are lawfully specified in ambient energy arrays. In contrast to more established informational variables—such as postural time-to-contact, which has been shown to exhibit lawful 1:1 mapping and clear relevance to affordance perception (Lee et al. 1998; Oudejans et al. 1996)—multifractality currently lacks a comparable specification framework. Whether multifractal fluctuations function as information for action, whether they are perceptually available, and whether organisms can become attuned to them are critical questions for future ecological research. These uncertainties do not diminish the value of multifractality for revealing the nested structure of control processes, but they caution against treating multifractality as an informational variable without first establishing its perceptual grounding and functional role in guiding action.

## The methodological circularity of internal model frameworks

The unfalsifiability of internal model theories isn’t just a methodological flaw—it’s a symptom of scientific decay: a reflex to convert failure into theory through endless post hoc patchwork, immunizing a collapsing framework against the very tests that should dismantle it.

### The circularity problem in testing representational hypotheses

Even if we entertain the possibility that neural systems implement something resembling internal models, testing this claim often amounts to little more than post hoc theoretical interpretation—where representational meaning is projected into neural data and then retrieved as if it had been objectively discovered. The foundational problem is this: internal model claims cannot be evaluated independently of the behavioral outcomes they were designed to explain in the first place (Bechtel 1998; Haselager et al. 2003; Ramsey 2007). The alleged representations are inferred from observed behavior, then retroactively used to “explain” that same behavior—a self-validating loop that mimics empirical reasoning while circumventing its constraints.

This circularity is baked into the dominant methodology: researchers record neural activity during sensorimotor tasks, fit a computational model that could plausibly generate the observed behavior, and then declare that the neural system must be implementing that model. The logic folds in on itself:

- The representational content of neural activity is never observed—it is inferred from the behavior the model was built to reproduce.
- The models are optimized to match that behavior, ensuring correlational fit by design.
- When predictions fail, models are padded with auxiliary hypotheses rather than rejected.

This is not empirical science—it is a self-affirming ritual of representational storytelling. And because the criteria for what counts as a representation are defined retroactively, the framework cannot fail on its own terms (Chemero 2009; Ramsey 2007). Any empirical pattern can be reinterpreted with enough mathematical duct tape.

### The unfalsifiability problem

Internal model frameworks suffer from a fundamental unfalsifiability problem—they can accommodate virtually any empirical outcome through post hoc theoretical adjustments. No matter how far neural activity or behavior diverges from the framework’s initial predictions, the theory survives—not by explanatory precision, but by strategic plasticity. When data contradict the model, we have collectively developed increasingly complex frameworks through one of three well-worn maneuvers:

- Adding hidden layers or free parameters to “better capture complexity”—effectively fitting the model to the noise (Körding and Wolpert 2004; Wolpert et al. 2011).
- Invoking ensembles of parallel models, with context-sensitive switching mechanisms that can be tuned post hoc to any outcome (Haruno et al. 2001; Wolpert and Kawato 1998).
- Attributing mismatches to insufficient training, incomplete adaptation, or unmodeled uncertainty—thereby deflecting theoretical critique onto experimental subjects (Lafleur et al. 2002; Krakauer et al. 2019).

**Fig. 5:**
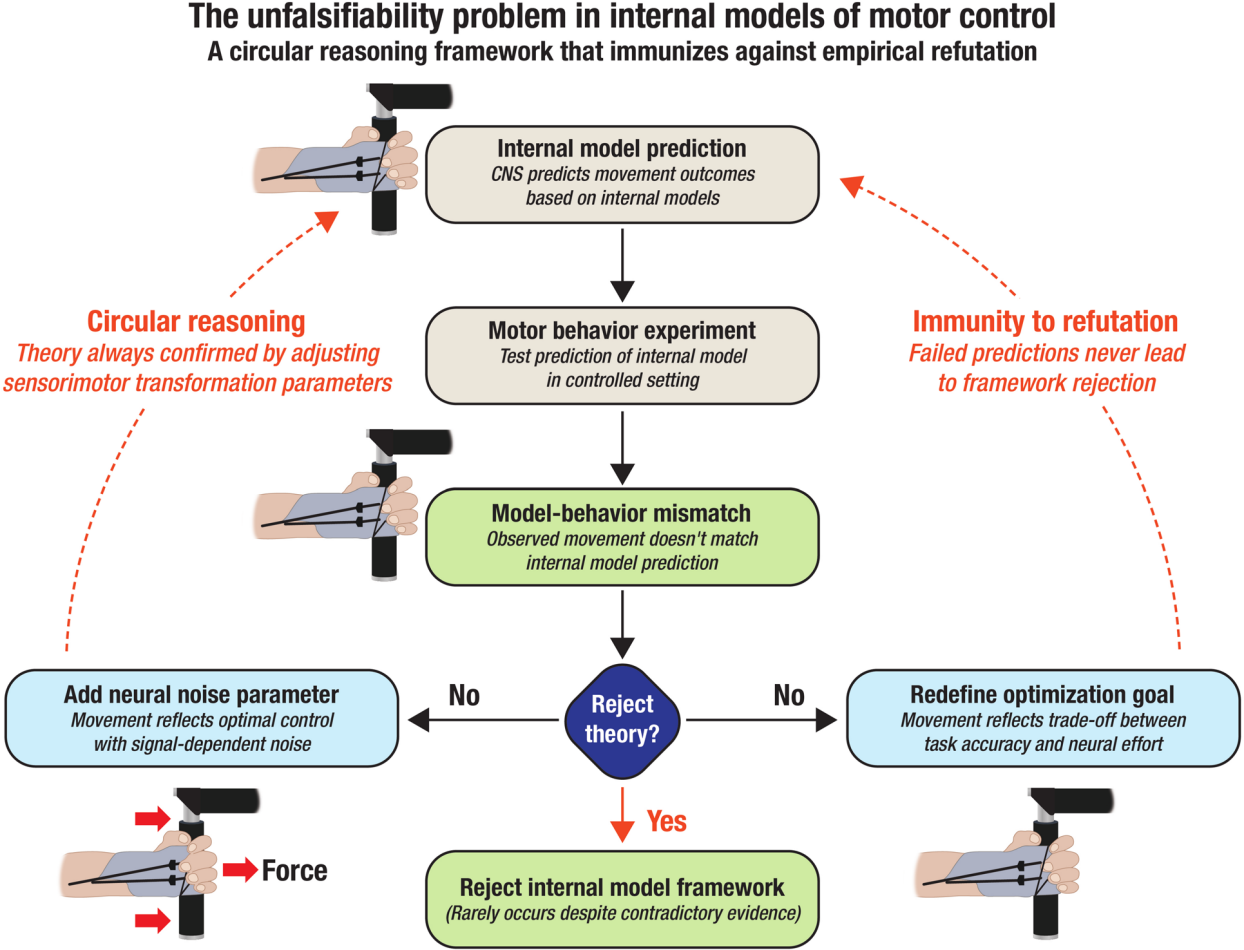
The unfalsifiability problem in internal models of motor control. This flowchart illustrates the methodological circularity that renders internal models of motor control effectively unfalsifiable. When experimental evidence contradicts model predictions (e.g., “observed movement doesn’t match internal model prediction”), researchers rarely reject the framework. Instead, they systematically protect the paradigm through post hoc adjustments—either by adding neural noise parameters (“movement reflects optimal control with signal-dependent noise”) or redefining optimization goals (“movement reflects trade-off between task accuracy and neural effort”). These adjustments create a self-reinforcing cycle that transforms empirical failures into theoretical successes, immunizing the framework against genuine falsification. This circular reasoning pattern reveals why internal model approaches persist despite repeated predictive failures: the theory automatically accommodates contradictory evidence through an endless proliferation of auxiliary hypotheses. This methodological structure violates standards of scientific testability, functioning more as mathematical rhetoric than as an empirically accountable mechanistic model of sensorimotor function

These adjustments are rarely framed as hypothesis revisions. Instead, they function as immunization strategies, protecting the framework from disconfirmation by ensuring it always has one more explanatory layer to deploy. The result is a theory that behaves less like a scientific model and more like a belief system—resilient not because it is accurate, but because it is unfalsifiable by design. For example, when participants adapt more slowly than predicted in visuomotor rotation tasks, researchers often cite “suboptimal model updating” or “recalibration lags” rather than reconsidering whether the system is model-based at all (Acerbi et al. 2014; Kording et al. 2007). When cerebellar activity fails to match predictions from forward model simulations, the discrepancy is not taken as evidence against internal models but as a cue to revise the simulation parameters. At no point is there a clear threshold for rejection (Fig. 5).

This theoretical elasticity ensures that internal models remain standing not because they succeed in predicting data, but because they are systematically insulated from failure. The problem is compounded by the abstractness of the core constructs—“representation,” “prediction,” “error signal”—which are not directly observable and lack operational criteria for empirical falsification (Bechtel 1998; Haselager et al. 2003; Ramsey 2007). Representations are inferred, not measured; error signals are posited, not isolated; predictive functions are assumed, not demonstrated.

In short, internal model frameworks are not structured to risk being wrong, and any theory that cannot be wrong cannot be right in any scientifically meaningful sense.

### Representation as mathematical rhetoric

The concept of internal models in sensorimotor control often functions less as an empirically grounded explanation and more as mathematical theater—a performance of precision that obscures conceptual incoherence (Chemero 2009; Ramsey 2007). Computational frameworks such as optimal control theory, Kalman filtering, Bayesian inference, and artificial neural networks offer elegant mathematical formalisms, but elegance is not evidence. These models are routinely calibrated to fit behavioral data, giving the impression of explanatory success while remaining agnostic—if not entirely silent—about how such computations are implemented, constrained, or even possible in neural tissue (Krakauer et al. 2017; Loeb 2012). This rhetorical function manifests in several recurring patterns:

- Behavioral fit is taken as validation, even when the underlying neural mechanisms remain speculative or incompatible with observed biology (Loeb 2012, 2021).
- Terms like “representation,” “prediction,” and “computation” serve as placeholder metaphors, invoking explanatory familiarity without providing mechanistic specificity (Anderson 2003; Barrett 2011).
- Models are tuned post hoc using free parameters (e.g., noise priors, cost functions, gain settings) that ensure good fit but lack empirical grounding, making the entire exercise indistinguishable from curve-fitting (Loeb 2012).

Consider the widespread use of optimal feedback control to explain human reaching behavior under uncertainty: researchers define a cost function after the fact to match the observed trajectory, then claim the brain must be minimizing that cost (Knill and Pouget 2004; Nagengast et al. 2010). But the cost function is not measured—it is inferred, and then used to confirm the very behavior it was designed to replicate. Similarly, Bayesian models of sensory integration adjust prior weights until they match empirical distributions, then report “Bayesian behavior” (Aller and Noppeney 2019; Deneve and Pouget 2004; Kording et al. 2004) as though it reflects internal implementation rather than statistical mimicry. What is missing is any independent validation: the priors and cost functions are not derived from biological measurement but reverse-engineered from the behavior they are supposed to explain, making the entire process indistinguishable from strategic curve-fitting. This kind of theoretical retrofitting turns modeling into a self-confirming rhetorical loop (Table 7; Bowers and Davis 2012; Jones and Love 2011). Instead of guiding empirical discovery, the formalism becomes a flexible justification—able to accommodate any pattern of behavior by adjusting unmeasured variables until the math fits. The result is a framework that feels scientific—thanks to its formalism—but fails to constrain scientific inference.

**Table 7:** Comparison of scientific modeling versus theoretical retrofitting

Scientific modeling	Theoretical retrofitting
Begins with a falsifiable hypothesis	Begins with a preferred model structure
Model is constrained by empirical outcomes	Model is modified to preserve fit regardless of outcome
Data that contradict predictions prompt revision or rejection	Contradictions are absorbed via new parameters or assumptions
Hypotheses are tested against independent observations	Model is calibrated using the very data it aims to explain
Failure indicates limits of the theory	Failure is reframed as incomplete learning or hidden complexity
Risk of falsification drives discovery	Immunity to falsification sustains belief

By providing a vocabulary that aligns with our intuitions about control and decision-making, internal model frameworks maintain their appeal even as they drift further from biological reality (Barrett 2011; Chemero 2009). But this appeal is esthetic, not explanatory. The framework’s greatest strength—its mathematical expressiveness and flexibility—is also its most dangerous flaw: it allows us to simulate what we do not understand, to fit models to data retroactively, and to mistake formal coherence for mechanistic truth.

It is essential to clarify that a model is not a theory. A model is a formal apparatus—an equation, simulation, or algorithm—that expresses assumptions made by a theory. It cannot select its own terms, variables, or structural relationships; only a causal theory can define those elements (Bailer-Jones 2009). Conflating model fit with theoretical confirmation not only obscures this distinction, but permits a dangerous reversal in which models appear to generate explanatory terms rather than formalize them (Frigg and Nguyen 2017). This sleight of hand allows researchers to treat models as ontologically real, even when they are merely computational conveniences. Similarly, the fact that empirical data (neural or behavioral) are consistent with a model’s outputs cannot be taken as evidence that the underlying biological system implements or depends upon that model. Correlation, even tight quantitative correspondence, is not causation (Glymour 2001; Pearl 2009). In many cases, these correlations are products of tuning model parameters to fit the data, not consequences of shared mechanism. Treating such alignment as confirmation of internal models collapses empirical science into rhetorical simulation.

## Alternative frameworks: beyond internal models

Moving beyond internal models opens new conceptual ground, where variability is not noise to suppress but a sign of adaptive potential, and stability arises through dynamic coupling rather than computational control. Performance is no longer judged by conformity to internal representations, but by flexible, context-sensitive coordination with the environment.

Clarifying model legitimacy beyond representationalism. In the *What this critique is not* section, I defend the utility of modeling while rejecting representationalist interpretations. But this raises a natural question: *If not internal models, what kinds of models are scientifically legitimate?* I suggest that models grounded in dynamical systems theory, ecological psychology, and non-representational predictive frameworks remain not only valid but vital. These approaches preserve explanatory rigor without resorting to speculative internal structures. They include, for example, dynamic field theory (Spencer and Ivry 2009), metastable coordination models (Tognoli and Kelso 2014), and predictive processing accounts that avoid representational commitments by emphasizing sensorimotor attunement rather than simulation (Bruineberg et al. 2018; Anderson 2003). Such models do not assume internal world reconstruction but instead describe lawful relations between neural, bodily, and environmental dynamics that unfold over time. They function not by positing internal surrogates of the world, but by tracing how functional behavior emerges from the system’s ongoing coordination with its context.

Toward biologically tractable modeling via nonlinear and multifractal dynamics. Moving beyond internal models opens the door to a new class of biologically tractable models grounded in nonlinear, non-Gaussian, and multifractal dynamics. These approaches do not merely tolerate variability—they treat its structure as meaningful. Rather than abstracting behavior into averaged trajectories or Gaussian residuals, multifractal and interaction-dominant models capture the rich, scale-dependent fluctuations that characterize real sensorimotor coordination (Ihlen 2012; Kelty-Stephen and Mangalam 2022). These statistical patterns are not implementation noise, but empirical signatures of the system’s adaptability, flexibility, and coupling to task-relevant information. Nonlinear models reveal how coordination emerges from metastable dynamics and soft-assembled synergies, with variability functioning as both a diagnostic and a mechanism of control (Kelso 1995; Latash 2012). In this view, modeling becomes useful not for postulating internal simulations, but for describing how structure, meaning, and function co-emerge from the organism’s engagement with its environment across time and scale.

### Ecological psychology: direct perception and affordances

Ecological psychology offers a radical alternative to internal model theories by proposing that perception-action systems operate through direct coupling with environmental information rather than through representational mediation (Gibson 1979; Turvey 1992). This approach reframes sensorimotor control in terms of the detection and utilization of affordances—opportunities for action that arise from the relationship between organismic capabilities and environmental properties (Chemero 2013; Reed 1996). Key principles of the ecological approach include:

- **Direct perception:** Organisms directly detect higher-order patterns in ambient energy arrays (optical, acoustic, haptic) without requiring inferential computation or representational reconstruction (Gibson 1979; Michaels and Carello 1981).
- **Affordances:** Environmental properties are perceived in terms of their action possibilities, creating a direct link between perception and action without requiring representational mediation (Chemero 2013; Turvey 1992).
- **Perception–action coupling:** Perception and action form a continuous cycle of mutual constraint, with perceptual information guiding action and action generating new perceptual information (Warren 2006; Wilson and Golonka 2013).
- **Information-based control:** Movement is controlled through the detection and use of task-specific information variables that directly specify appropriate actions without requiring internal models (Fajen 2013; Warren 2006).

This ecological framework has successfully explained a wide range of sensorimotor phenomena without invoking internal models or representations:

- Tau-coupling in timing actions such as catching, hitting, or braking, where movement is directly regulated by optical variables specifying time-to-contact (Lee et al. 1998).
- Visually guided locomotion, where path selection and obstacle avoidance emerge from the detection and use of optical flow patterns without requiring internal world models (Fajen and Matthis 2013; Warren 2006).
- Dynamic touch, where object properties are directly perceived through exploratory movements that generate specific patterns of sensory invariants (Carello et al. 2012; Turvey and Carello 2011).
- Perceptual learning, where sensitivity to action-relevant information variables improves through exploration and practice without requiring explicit representational updating (Fajen 2013; Jacobs and Michaels 2007).

The ecological approach shifts focus away from computational internal models toward how organisms directly detect and respond to structured information in their environment (Gibson 1979). It emphasizes the continuous, real-time coupling of perception and action, where behavior emerges from lawful organism–environment interactions rather than from internal representations (Turvey 1992). This framework offers a more parsimonious, robust, and biologically grounded explanation for a wide range of sensorimotor phenomena (Warren 2006).

It is important to acknowledge that recent developments within ecological and enactive traditions have not been uniform in their commitment to Gibson’s original formulation of direct perception. Notably, Chemero (2006), Chemero (2009) and Bruineberg and colleagues (2018; 2019) have advocated for a broader notion of *general ecological information*, which explicitly loosens the requirement for lawful, 1:1 specification in ambient energy arrays. In their view, many affordances may not be lawfully specified at all and instead require internal, inferential processes—often framed in terms of Bayesian priors, internal state estimation, or sensitivity to statistical regularities. While this theoretical expansion has enriched ecological theory and fostered connections with predictive processing, it marks a shift away from Gibsonian commitments. Crucially, it risks reinstating the inferential and representational architecture that ecological psychology originally sought to eliminate.

By contrast, the present manuscript uses the term *direct perception* in its strict Gibsonian sense: as perceptual access grounded in the lawful specification of environmental properties in structured energy arrays. On this view, information is neither constructed nor internally simulated; it is detected through attunement to higher-order invariants available in the organism’s ecological niche. This definition excludes any need for inferential supplementation. The commitment here is categorical: wherever internal inference is invoked—whether Bayesian or not—the theoretical scaffolding collapses back into the representational logic that underwrites internal model frameworks. In this regard, the present critique aligns with recent arguments by Stoffregen and Wagman (2025), who argue that the turn toward general ecological information blurs the distinction between direct and indirect perception and thereby undermines the explanatory integrity of ecological psychology.

My position also extends from my broader critique of inference-based cognitive theories. In a recent deconstruction of the Bayesian brain hypothesis (Mangalam 2025), I argue that Bayesian logic, despite its formal elegance, often functions as a rhetorical backdoor for representational commitments. This concern is not merely conceptual but foundational: relaxing the criterion of lawful specification in favor of internal estimation risks erasing the very conceptual boundary that separates ecological theory from the internalist architectures it was meant to displace.

### Dynamical systems theory: self-organization and emergence

Dynamical systems theory offers another powerful alternative to internal model frameworks by focusing on how coordinated movement emerges from the intrinsic dynamics of coupled systems without requiring representational control (Kelso 1995; Thelen and Smith 1994). This approach reframes sensorimotor control in terms of self-organizing dynamics, pattern formation, and nonlinear interactions across scales (Haken et al. 1985; Kelso 2009). Key principles of the dynamical systems approach include:

- **Self-organization:** Coordinated patterns emerge spontaneously from the intrinsic dynamics of the system without requiring centralized control or representation (Haken et al. 1985; Kelso 1995).
- **Attractor dynamics:** Behavior is understood in terms of attractor states and phase transitions, with coordination patterns forming stable attractors that can be destabilized and reformed as control parameters change (Haken 2009; Kelso 1995).
- **Multiscale interactions:** Coordination emerges from nonlinear interactions across multiple spatial and temporal scales, from neural dynamics to biomechanics to environmental constraints (Ihlen and Vereijken 2013; Van Orden et al. 2003).
- **Metastability:** Adaptive behavior operates in a metastable regime near critical points, balancing stability and flexibility to enable context-sensitive coordination (Kelso 1995; Tognoli and Kelso 2014).

This dynamical framework has successfully explained a wide range of sensorimotor phenomena without invoking internal models or representations:

- Bimanual coordination, where stable patterns emerge from the coupling dynamics of oscillatory components rather than from representational control (Haken et al. 1985).
- Postural control, where stability emerges from the continuous coupling of multiple subsystems without requiring explicit internal models of body dynamics (Balasubramaniam and Wing 2002; Riley and Turvey 2002).
- Motor development, where new skills emerge through exploration of dynamic stability properties rather than through the acquisition of representational models (Smith and Thelen 2003; Thelen and Smith 1994).
- Perceptuomotor learning, in which adaptation reflects gradual and experience-driven changes in the attractor landscape of the entire organism–environment system, rather than discrete updates to internal representations (Kostrubiec et al. 2012; Zanone and Kelso 1992).

The dynamical systems approach shifts attention from internal computations to how coordination patterns emerge through the intrinsic dynamics of interacting biological and environmental systems. It highlights self-organization and emergence as core principles, viewing behavior as the product of system-wide coupling rather than central control. This perspective offers a simpler and more mechanistically grounded account of sensorimotor behavior across contexts (Kelso 1995; Thelen and Smith 1994).

### Synergetics and coordinative structures

Building on dynamical systems principles, the concepts of synergetics and coordinative structures provide a specific framework for understanding how complex movements are organized without requiring explicit representational control (Haken 2009; Turvey 1990). This approach focuses on how functional groupings of neural, muscular, and skeletal elements are temporarily assembled to serve specific task demands (Latash 2008; Turvey 1992). Key principles of the synergetic approach include:

- **Coordinative structures:** Functional units that temporarily constrain the degrees of freedom of the system to serve specific task demands without requiring explicit computational control (Kelso 1991; Turvey 1990).
- **Soft assembly:** Coordination patterns are softly assembled in response to task constraints rather than hardwired or pre-programmed, allowing for contextual flexibility (Thelen 1995; Turvey 2007).
- **Degeneracy:** Multiple coordinative configurations can achieve the same functional outcome, creating robust flexibility without requiring optimal control (Edelman and Gally 2001; Latash 2012).
- **Uncontrolled manifold:** Task-relevant variables are stabilized while task-irrelevant variables are allowed to fluctuate, creating a solution space rather than a single optimal trajectory (Latash 2008; Scholz and Schöner 1999).

This synergetic framework has successfully explained a wide range of sensorimotor phenomena without invoking internal models or representations:

- Multi-joint movements, where coordinative structures temporarily constrain degrees of freedom to create functional movement patterns without requiring explicit computational control (Kelso 1991; Turvey 1990).
- Force production tasks, where stability in task-relevant dimensions coexists with variability in task-irrelevant dimensions, reflecting functional synergy rather than computational optimization (Latash 2008; Scholz and Schöner 1999).
- Adaptability to perturbations, where coordinative reorganization occurs rapidly in response to changing constraints without requiring explicit model updating (Latash 2008; Peterka 2002).
- Motor equivalence, where consistent outcomes are achieved through flexible means, reflecting synergetic organization rather than representational control (Kelso 1991; Latash 2012).

The synergetic approach shifts emphasis from internal computation to how functional movement patterns emerge through the soft assembly of task-specific synergies. It explains coordination as arising from the interaction of contextual constraints, body dynamics, and the degeneracy of multiple movement solutions. This framework removes the need for explicit representations and provides a more flexible, parsimonious account of sensorimotor behavior (Kelso 1991; Turvey 1990).

### Predictive processing without representation

Even within frameworks that emphasize prediction, alternatives to representational internal models have emerged that focus on dynamic coupling rather than computational representation (Bruineberg et al. 2018; Gallagher 2017). These approaches reframe predictive dynamics in terms of embodied attunement to environmental regularities rather than internal model-based computation (Anderson 2003; Warren 2006). Key principles of non-representational predictive frameworks include:

- **Anticipatory dynamics:** Systems become attuned to environmental regularities through dynamic coupling rather than through explicit representation or computation (Stepp and Turvey 2010; Turvey 1992).
- **Sensorimotor contingencies:** Knowledge of sensorimotor relationships is embedded in the system’s dynamics rather than stored as explicit models or representations (O’regan and Noë 2001; van Doorn et al. 2007).
- **Metastable perception–action systems:** Prediction emerges from the metastable dynamics of coupled perception–action systems without requiring explicit representational mediation (Kelso 2012; Warren 2006).
- **Radical predictive processing:** Predictive dynamics can be reframed in terms of system attunement to environmental regularities without requiring the strong representational claims of traditional predictive coding (Bruineberg et al. 2018; Kirchhoff et al. 2018).

Ecological accounts emphasize that affordances are not descriptions of what is happening, or records of what has happened—they are structured possibilities for what could happen. Affordances are fundamentally prospective: they refer to the potential for action available to an organism in the present, based on the dynamic relation between its capabilities and the surrounding environment (Fajen 2007; Jansen and Fajen 2024). Crucially, this prospectivity does not imply internal prediction or representational inference. This distinction is often obscured because prospectivity and prediction both concern the future, but they differ fundamentally in mechanism. If affordances are lawfully specified in ambient arrays, they can be directly perceived as opportunities for future action—without requiring the nervous system to simulate or anticipate internal futures. What looks like anticipatory behavior under internal model frameworks may instead reflect real-time attunement to lawful, future-facing information structures. Thus, even under conditions that appear to demand “prediction,” the ecological approach offers an alternative grounded in present-time specification and perception.

These non-representational predictive frameworks have successfully explained a wide range of sensorimotor phenomena without invoking internal models in the traditional sense:

- Sensorimotor adaptation, where systems become attuned to novel contingencies through exploration and dynamic coupling rather than through explicit model updating (Banakou et al. 2013; van Doorn et al. 2007).
- Skilled performance, where anticipatory dynamics emerge from extensive practice that attunes the system to task-relevant information without requiring explicit representations (Anderson 2003; Clark 2015).
- Perceptual presence, where the sense of engaging with real objects emerges from mastery of sensorimotor contingencies rather than from representational reconstruction (Noë 2004; O’regan and Noë 2001).
- Error correction, in which adjustments to movement patterns emerge from the intrinsic sensitivity of coupled perception–-action systems to environmental and task-related perturbations, without requiring explicit comparisons to internal models or stored representations (Bardy et al. 2002; Huys et al. 2003).

These frameworks explain the anticipatory and adaptive features of sensorimotor behavior without relying on the strong representational assumptions central to internal model theories. Rather than invoking internal simulations, they account for prediction as emerging from the continuous, real-time interplay between an organism and its environment. By emphasizing dynamic coupling, perceptual attunement, and sensorimotor contingencies, these approaches offer more parsimonious and empirically grounded alternatives to representational accounts.

It is important to acknowledge that ecological and dynamical frameworks, like internal model theories, are guided by foundational conceptual motifs that shape their epistemic commitments. As Raja (2024) argue, motifs such as “distributed control” or “resonance” function not as testable hypotheses but as orienting commitments that structure explanation. In this sense, ecological psychology is no less theory-laden than its representationalist counterpart. However, there is a meaningful distinction between the motifs of direct perception and self-organization—anchored in observable organism–environment coupling—and the motif of inferential computation, which tends to posit internal mechanisms that are not directly accessible to empirical manipulation. The present critique does not claim that ecological frameworks are immune to empirical vulnerability, but rather that they invite different forms of testability—focused on system-level behavior, perturbation-based dynamics, and relational invariants. The aim is not to replace one infallible framework with another, but to realign the theoretical landscape around biologically grounded, empirically tractable principles of coordination and control.

## Implications and applications: moving beyond internal models

We must abandon the seductive fiction of internal models and face what they obscure: sensorimotor control is not computed but lived, emerging from the irreducible entanglement of organism and environment.

### Implications for neuroscience and motor control

Abandoning internal model frameworks demands more than a theoretical course correction—it requires neuroscience to confront the consequences of decades spent chasing metaphors. For years, motor control research has fixated on locating the neural substrates of forward and inverse models—attempts to find cerebellar circuits that predict future states, or cortical regions that calculate desired trajectories. But these quests have yielded little more than noisy correlations and ambiguous activation maps. The promised mechanisms remain speculative, while the models themselves are retrofitted to accommodate whatever the data happen to show. The alternative is not to abandon modeling but to stop mistaking engineering metaphors for biology. Instead of asking whether the brain simulates the body, we must ask how brain activity entrains with bodily and environmental dynamics in real time. Several research shifts follow:

- Investigate how neural oscillations synchronize with motor output, environmental rhythms, and sensory inputs across multiple timescales—moving beyond static mappings to dynamic coordination (Kelso 1995; Thompson 2010).
- Study how metastability in neural systems supports context-sensitive transitions in movement and behavior, such as spontaneous switching in bimanual coordination (Tognoli and Kelso 2014).
- Reinterpret cerebellar activity not as simulating sensory consequences, but as a timing scaffold for fine-tuning movement, perception, and cognition (Ivry and Spencer 2004; Spencer and Ivry 2009).
- Abandon reductionist visuomotor paradigms that isolate sensory input and motor output in favor of closed-loop, ecologically valid tasks where perception and action unfold together (Gibson 1979; Maselli et al. 2023).

Consider how internal model theories have shaped interpretations of saccadic eye movement: the idea that the brain uses an efference copy to predict the expected retinal displacement during a saccade is intuitively compelling—but likely unnecessary. Evidence shows that visual stability may arise from the dynamics of reafferent feedback and sensorimotor timing rather than prediction (Wurtz 2008). Similarly, in studies of reaching and motor adaptation, Bayesian estimation and forward modeling are routinely invoked to explain trial-by-trial variability. Yet this variability often exhibits lawful, multifractal structure and appears to reflect the constraints and affordances of the task environment, rather than internal noise or representational uncertainty (Kelty-Stephen et al. 2021; Kelty-Stephen and Mangalam 2022). In both cases, internal model interpretations impose a layer of abstraction that adds complexity while obscuring the direct, dynamical interactions driving behavior.

This critique is not intended to dismiss the valuable insights or methodological innovations that have emerged from internal model frameworks. Many researchers working within that tradition have advanced our understanding of motor behavior, and their efforts reflect serious engagement with the complexity of sensorimotor control. Rather, the present argument invites a re-evaluation of foundational assumptions and encourages greater openness to alternatives that may offer more biologically grounded accounts. Ecological and dynamical approaches are not offered here as dogmatic replacements but as urgently needed expansions—frameworks that can coexist in critical dialogue with internal model theories while pushing the field toward greater explanatory depth. The goal is not to shut down debate, but to refocus it: away from abstract simulations and toward the embodied, real-time coordination that defines how organisms actually move.

What is at stake here is not merely conceptual clarity, but the long-term trajectory and integrity of neuroscience itself. Too much research funding, institutional attention, and intellectual energy continue to be poured into chasing theoretical ghosts—hypothesized model components that have never been biologically observed or mechanistically grounded. This misplaced focus perpetuates a fiction that distorts both methodology and interpretation. Neuroscience must urgently redirect its attention toward how neural systems function in real-time, dynamic coordination with the body and environment—not as internal simulators, but as embedded participants in action. The payoff is not only conceptual coherence, but a science capable of revealing how real movement, perception, and adaptation unfold in living systems.

### Implications for rehabilitation and clinical practice

Moving beyond internal model frameworks carries serious consequences for how rehabilitation is conceptualized and delivered. Traditional rehabilitation often aims to restore a pre-defined “normal” movement pattern, implicitly grounded in the belief that the nervous system is working to reestablish an optimal internal model. This approach pathologizes variability and centers treatment on conformity to averaged templates, rather than on individualized function (Krakauer and Carmichael 2022; Levin et al. 2016). But this model-centric logic has yielded limited gains in real-world recovery. A non-representational, dynamical perspective instead emphasizes adaptability, exploration, and the reorganization of coordination under new constraints (Harbourne and Stergiou 2009; Newell 2003). Specific clinical shifts include:

- Encouraging movement exploration and discovery of functional solutions rather than imposing “ideal” trajectories (Harbourne and Stergiou 2009; Newell 2003).
- Designing therapy environments that manipulate task and environmental constraints to support emergent coordination patterns (Newell 1986; Wu et al. 2000).
- Viewing variability not as noise but as evidence of adaptive potential and a means for enhancing robustness across contexts (Harbourne and Stergiou 2009; Stergiou and Decker 2011).
- Prioritizing patient-specific goals and abilities over normative templates derived from computational models of “optimal” function (Krakauer and Carmichael 2022; Levin et al. 2016).

This reframing shifts rehabilitation away from chasing fictive internal architectures and toward fostering real-world adaptability. It prioritizes coordination, constraint, and context over control, correction, and computation. Most importantly, it restores the lived experience of movement to the center of clinical care.

### Implications for robotics and artificial intelligence

Internal model frameworks have deeply shaped the fields of robotics and artificial intelligence, where control-theoretic architectures and representational processing remain entrenched paradigms (Brooks 1991; Pfeifer et al. 2007). This influence has produced machines that are computationally elaborate yet behaviorally brittle—impressive in simulation, but fragile in the unpredictable messiness of the real world. Clinging to internal model logic has yielded robots that can solve maze-like puzzles in controlled settings, yet still struggle with the fluid, adaptive coordination that even a crawling infant executes with ease. In contrast, emerging approaches rooted in ecological dynamics, morphological computation, and self-organization promise more robust, flexible, and environmentally attuned systems (Chemero 2009; Pfeifer et al. 2012). These embodied strategies offer not just alternatives, but correctives to the limitations of internalist design. Key implications for robotics include:

- Designing morphologically intelligent systems where adaptive behavior arises from the interaction between body shape, material properties, and environmental constraints—minimizing the need for centralized control (Comoretto et al. 2025; Pfeifer et al. 2007, 2012).
- Developing soft robots that harness passive dynamics and material intelligence to achieve coordination without complex internal modeling (Ha et al. 2018; Rus and Tolley 2015).
- Shifting from model-heavy architectures to learning systems that discover and use information variables tied to specific tasks, not general world reconstruction (Morimoto and Doya 2001; Sutton and Barto 1998).
- Creating systems that operate in metastable regimes near critical points, enabling flexibility and robustness without over-specifying behavior in advance (Aguilera et al. 2013; Prokopenko 2013).

This reframing urges robotics to abandon the fantasy of digital omniscience and instead embrace physical intelligence—behavior that emerges not from internal simulations, but from real-time interaction with the world (Pfeifer and Bongard 2006). Intelligence is not embedded in code, but arises from the structure of engagement, shaped by morphology, materials, and context. The payoff is not just more robust machines, but a redefinition of what it means to design something that behaves intelligently.

### Implications for education and skill acquisition

Abandoning internal model frameworks demands a major shift in how we understand learning. Traditional instructional approaches treat skill acquisition as the internalization of idealized templates, emphasizing error correction and conformity to “optimal” movement patterns—a logic rooted in representational assumptions (Newell 1985; Schmidt 1975). This model-centric pedagogy pathologizes variability and reduces learners to processors of instructions, rather than agents discovering viable solutions through interaction with their environment. Decades of constraints-led research have shown this to be both biologically implausible and pedagogically limiting (Davids et al. 2008; Newell 1986). Worse, it fosters dependence on external correction rather than cultivating the learner’s ability to adapt, perceive, and solve problems in dynamic contexts. A dynamical, ecological approach reframes learning as the attunement of perception-action systems to information in context. This shift transforms the educator’s role: from instructor to environment designer, from dispenser of commands to architect of conditions that support functional exploration. Specific strategies include:

- Designing practice environments that manipulate constraints to elicit adaptive solutions, rather than enforcing uniform movement patterns (Chow et al. 2021; Newell 1986).
- Encouraging exploration and movement variability as productive, rather than treating them as noise (Dhawale et al. 2017; Stergiou and Decker 2011).
- Directing attention to meaningful information in the task environment, rather than teaching abstract rules divorced from real-time interaction (Jacobs and Michaels 2007; Warren 2006).
- Embracing nonlinear pedagogy that supports individual trajectories of learning and rejects one-size-fits-all progression (Chow et al. 2021; Newell 2003).

This reframing returns agency to the learner and ecological relevance to the learning environment. It privileges dynamic coordination, constraint-based exploration, and self-organization over rote repetition, internal representation, or abstract “error correction.” The result is not just better pedagogy, but a learning science that actually reflects how real bodies learn to move in real, variable, and task-rich worlds.

### What this critique is not

This section addresses common misreadings of the argument and clarifies what this critique does—and does not—reject. It does not argue against the use of mathematics in the study of biological systems, nor does it dismiss the value of simulations, data-driven inference, or algorithmic analysis. Modeling remains essential to scientific inquiry, and abstractions are necessary for navigating complex systems. What this critique challenges is the uncritical persistence of a particular kind of representationalism—one that conflates metaphor with mechanism and treats internal models not as heuristic or modeling devices, but as literal, neurobiological realities. This assumption often masquerades as explanatory rigor while relying on constructs that evade falsification, lack neural grounding, and collapse under philosophical scrutiny.

Many current models labeled as “internal” may, in fact, reflect emergent, interaction-dominant dynamics rather than the storage and manipulation of explicit, neurally encoded representations. When disentangled from strong representational claims, many such models may still offer empirical utility, predictive coherence, and formal elegance as compact descriptions of system behavior. But their interpretation must shift fundamentally: from descriptions of hypothetical computational architectures to approximations of system-level dynamics that unfold within the real-time, reciprocal coupling between organism and environment.

This critique therefore invites re-interpretation, not demolition. Models can remain valuable scientifically if they are understood not as literal blueprints of neural computation, but as approximations of embodied, temporally extended, and context-sensitive processes. Their usefulness lies not in mapping hypothetical internal constructs, but in capturing the relational dynamics that emerge through continuous coupling between organism and environment. Modeling, in this view, becomes a way to approximate emergent coordination rather than to represent internal simulation. Regrounded in this way, modeling becomes an instrument for revealing dynamic relational processes—not a speculative mirror of internal computation.

#### Anticipating objections

To further disambiguate, I outline five common misunderstandings and clarify the position of this manuscript in relation to each:

- **This is not a rejection of modeling.** I value formal modeling as a critical method in science. What I critique is the conflation of certain models—especially those labeled as “internal”—with literal mechanisms of neural function, without sufficient empirical justification.
- **This is not anti-computation.** I do not oppose computational thinking or analysis per se. I challenge the uncritical transfer of control-theoretic architectures from engineering into biology, where assumptions fail to hold.
- **This is not an attack on mathematics.** Mathematical frameworks are indispensable for formalizing hypotheses and generating predictions. The issue lies not in math, but in how mathematical abstractions are interpreted—particularly when they are reified into claims about internal structures that remain unobserved.
- **This is not a denial of prediction.** Biological systems often exhibit anticipatory dynamics. But prediction need not imply internal simulation or representation. Anticipation can emerge from real-time, dynamical coupling between system and environment.
- **This is not mere philosophy.** This critique engages philosophical concepts, but it is grounded in empirical anomalies, methodological circularity, and biologically motivated alternatives.

These clarifications are not hedges—they are part of our broader argument that effective modeling demands conceptual discipline, empirical constraint, and interpretive humility.

## Conclusion: beyond the myth of internal models

When we discard the illusion of internal models, we don’t find chaos—we find the real order of life: not computation, but coordination; not optimization, but exploration; not code, but embodiment.

Internal model frameworks in sensorimotor control represent one of the most consequential theoretical failures in modern neuroscience—a half-century detour into computational fantasy that has systematically misdirected empirical inquiry. They rest on outdated philosophical assumptions, resist empirical falsification, and thrive on methodological circularity. What once promised explanatory rigor now functions largely as a metaphor—useful for engineering, but corrosive to biology. This critique leads to several inescapable conclusions:

- Internal models resurrect a covert Cartesianism, separating controller from controlled and distorting the fundamentally entangled nature of perception and action.
- The supposed neural implementations of internal models remain a speculative fiction, sustained more by disciplinary habit than empirical evidence.
- The representational scaffolding of internal models collapses under the weight of its own contradictions: homunculi, symbol grounding, and inference machines imagined into neural tissue.
- The multiscale, nonlinear, and non-Gaussian dynamics of biological movement are not noise to be filtered out but signal—the signature of a system that does not compute, but self-organizes.
- Alternative paradigms—ecological dynamics, constraint-based models, synergetics—offer empirically grounded and biologically coherent ways of understanding adaptive behavior without resorting to internal fictions.

The time for polite academic disagreement has passed. Internal model frameworks are not merely wrong—they are anti-scientific, immunized against falsification, and sustained by a combination of intellectual laziness, career incentives, and institutional momentum. They represent a cautionary tale of how entire scientific communities can become ensnared in intricate, self-reinforcing theoretical architectures—elaborate constructions that persist not because they illuminate biological reality, but because they justify their own continued existence.

To move beyond internal models is not a loss but a liberation. It frees us from the reductive impulse to reverse-engineer brains into machines they never were, and instead invites us to study biological movement as it truly is: richly variable, deeply context-dependent, and dynamically shaped by the ongoing interplay of organism, environment, and task constraints. Clinging to internal models not only narrows our scientific vision but also flattens the very complexity we should be striving to understand, replacing embodied dynamics with abstract fictions that explain less than they assume.

The myth of internal models is not just a conceptual misstep—it is a symptom of a deeper failure to confront the limits of our metaphors and the comfort they provide. Just as past centuries projected theological order onto nature, contemporary neuroscience projects computational logic onto neural systems that owe far more to evolutionary history, physical constraint, and embodied interaction than to design or programming. By shedding these illusions, we can begin to see biological movement not as a process to be decoded or reverse-engineered, but as a dynamic, multiscale phenomenon emerging from the reciprocal constraints of body, task, and environment—one that demands explanation over simulation, engagement over abstraction, and understanding over engineering metaphor.

What is at stake in this critique extends beyond theoretical nuance. The persistence of internal model frameworks risks misdirecting funding priorities, funneling resources toward speculative constructs instead of empirically grounded inquiry. It narrows the horizons of scientific investigation, crowding out alternative frameworks rooted in embodiment, dynamics, and ecological interaction. It shapes curricula, experimental paradigms, and scientific worldviews around a metaphor that often obscures more than it reveals. And in doing so, it commits a generation of researchers and students to refining an explanatory architecture whose empirical support remains elusive and whose philosophical coherence is, at best, unstable.

Future historians of science will marvel at how an entire generation of researchers convinced themselves that brains compute with internal models—a collective delusion sustained by methodological circularity, career incentives, and the seductive appeal of engineering metaphors applied where they fundamentally do not belong.

For computational neuroscientists, this critique urges a fundamental re-evaluation of what it means to model biological systems—advocating a move away from abstract, disembodied simulation toward empirically constrained, dynamical frameworks that remain grounded in biological reality. For experimentalists and clinicians, it suggests more robust ways to interpret variability, adaptation, and coordination without relying on unobservable internal architectures or speculative cognitive surrogates. For philosophers of science, it offers a cautionary tale about the inertia of metaphor and the profound epistemological risks of conceptual overextension—especially when metaphors, originally intended as heuristics, harden prematurely into method, guiding not just interpretation but the very design of experiments, models, and theories. And for the next generation of movement scientists, it opens a path forward—grounded in embodiment, environmental interaction, and the multiscale complexity of sensorimotor behavior. Across all domains, the argument demands an immediate and uncompromising shift from speculative representational frameworks to process-based understanding of how biological coordination actually unfolds in time, space, and context.

## Data Availability

Data sharing does not apply to this article as no new data were created or analyzed in this study.
